# Spa therapy with physical rehabilitation is an alternative to usual spa therapy protocol in symptomatic knee osteoarthritis

**DOI:** 10.1038/s41598-020-67436-1

**Published:** 2020-07-03

**Authors:** Anne-Christine Rat, Damien Loeuille, Amandine Vallata, Lorraine Bernard, Emmanuel Spitz, Alexandra Desvignes, Michel Boulange, Jean Paysant, Francis Guillemin, Isabelle Chary-Valckenaere

**Affiliations:** 1 EA 4360 APEMAC, Université de Lorraine, 54500 Nancy, France; 2 CHRU-Nancy, INSERM, Université de Lorraine, CIC 1433 Clinical Epidemiology, Nancy, France; 3 Inserm CIC-EC 1433, Nancy, France; 4 Department of Rheumatology, Nancy University Hospital, 54511 Vandoeuvre-les-Nancy, France; 5 IMoPA, Université de Lorraine, Nancy, France; 6 Université de Lorraine, Nancy, France; 7 Regional Institute for Physical and Rehabilitation Medicine-Louis Pierquin Center of Nancy, 54500 Nancy, France

**Keywords:** Rehabilitation, Osteoarthritis

## Abstract

The objective of the study was to demonstrate the non-inferiority of low-frequency spa therapy combined with rehabilitation (Spa-rehab) versus standard spa therapy at 6 months for symptomatic knee osteoarthritis (KOA). A prospective, randomized, monocenter, non-inferiority trial with recruitment of community-based symptomatic KOA patients was performed. Standard spa therapy comprised standardized spa treatment, 6 days a week for 3 weeks, and Spa-rehab therapy comprised spa sessions, 3 days a week for 3 weeks, followed by a dedicated rehabilitation program, 3 days a week for 3 weeks. The primary endpoint was achieving at 6 months a minimal clinically important improvement (MCII) for pain on a visual analog scale and/or an MCII for function on the WOMAC index and no knee surgery (composite MCII). Secondary endpoints were composite MCII at 3 months and achieving a Patient Acceptable Symptom State (PASS) for pain and function at 3 and 6 months. Among 283 patients included, 145 were allocated to standard spa therapy and 138 to Spa-rehab therapy. We could not demonstrate the non-inferiority of Spa-rehab therapy for the primary endpoint: difference for responders − 0.08 [90% CI (− 0.18 to 0.02), *p* = 0.14]. However, the difference test between the groups was not significant (*p* = 0.18). Spa-rehab therapy was not inferior to standard spa therapy for the composite MCII at 3 months or the PASS at 3 and 6 months. Spa-rehab therapy can reasonably be proposed to patients with symptomatic KOA. This protocol may be more cost-effective than standard spa therapy and avoid absenteeism from work and accommodation costs for patients who live close to a centre.

## Introduction

Estimates from the 2017 Global Burden of Disease study highlight that hip and knee osteoarthritis (KOA) is one of the leading causes of global disability^1^. Its population-based prevalence in western countries is increasing^2^ and estimates of symptomatic KOA prevalence range from 5.4 to 29.8%^3,4^.

Several professional recommendations for KOA care (European League Against Rheumatism^5^, American College of Rheumatology^6^ and the Osteoarthritis Research Society International^7,8^) highlight the role of non-pharmacological approaches^9,10^, including weight reduction, education, aerobics, muscle strengthening and water-based exercises and spa therapy^5^. In a systematic review, aerobic, resistance, and performance exercises had similar effects on reducing pain^11^. However, the benefit seemed to be short (< 6 months)^11,12^ and most clinical trials assessing the impact of physical activity (and especially land-based exercise) have a short-term follow-up ranging from 6 to 12 weeks^13^. High-quality evidence indicates that land-based therapeutic exercise provides short-term benefit in terms of reduced knee pain, and moderate-quality evidence shows improvement in physical function among people with knee OA^6^. The magnitude of the treatment effect would be considered moderate (immediate) to small (2–6 months) but comparable with estimates reported for non-steroidal anti-inflammatory drugs^6^.

An approach combining exercises to increase strength, flexibility and aerobic capacity seems to be most effective in managing KOA^14^.

Several randomized controlled trials (RCTs) have assessed the efficacy of spa therapy for KOA^9,15–17^. The estimated effect size (ES) for spa therapy on pain to support recommendations was 0.46 (0.17–0.75)^18^, which is moderate but comparable than that for pharmacologic treatments. In a recent systematic review, different types of spa therapy were considered as effective to reduce pain, nonsteroidal anti-inflammatory drug consumption, and functional limitation and to improve quality of life^19^. However, because spa therapy (defined as thermal baths containing mineral waters and exercising in a health near a mineral or hot spring) is a complex intervention, analyzing its efficacy is difficult and the quality of evidence is only fair^10^.

Efficacy has been demonstrated with some spa protocols; this is the case for the usual and only reimbursed protocol in France; however, patients often complain of fatigue and the inability to follow such intensive and long spa stays. Moreover, spa protocols for OA have seldom been compared. An outpatient protocol, less intensive, but for a longer time, combining usual spa therapy and rehabilitation care, is an innovative format and could be more appropriate than standard spa therapy for some patients.

In this RCT, we hypothesized that the effect of this modified spa therapy protocol is not inferior to the usual standard protocol but is not clinically superior. The aim was to develop an intervention better suited to patients who do not want to spend 3 weeks off work. Indeed, non-pharmacological treatment and exercise recommendations to patients should focus on the patient’s preferences and access, both of which may be important barriers to participation. If a patient does not find a certain form of care acceptable or cannot afford it, he/she is not likely to pursue this treatment.

The primary objective of the study was to demonstrate the non-inferiority at 6 months of a combined spa therapy and rehabilitation protocol consisting of spa sessions every other day for 3 weeks, followed by rehabilitation sessions 3 days a week for 3 weeks (Spa-rehab therapy) versus the current standard spa protocol that is reimbursed in France: daily spa sessions for 3 consecutive weeks (standard spa therapy). Secondary objectives were to demonstrate the non-inferiority of Spa-rehab therapy versus standard spa therapy at 3 months, to describe the evolution of pain, function, quality of life (QoL), fatigue, drug consumption and satisfaction with the 2 therapies, and to determine the predictive factors of response to therapy at 6 months.

## Patients and methods

### Study design

**Figure 1:**
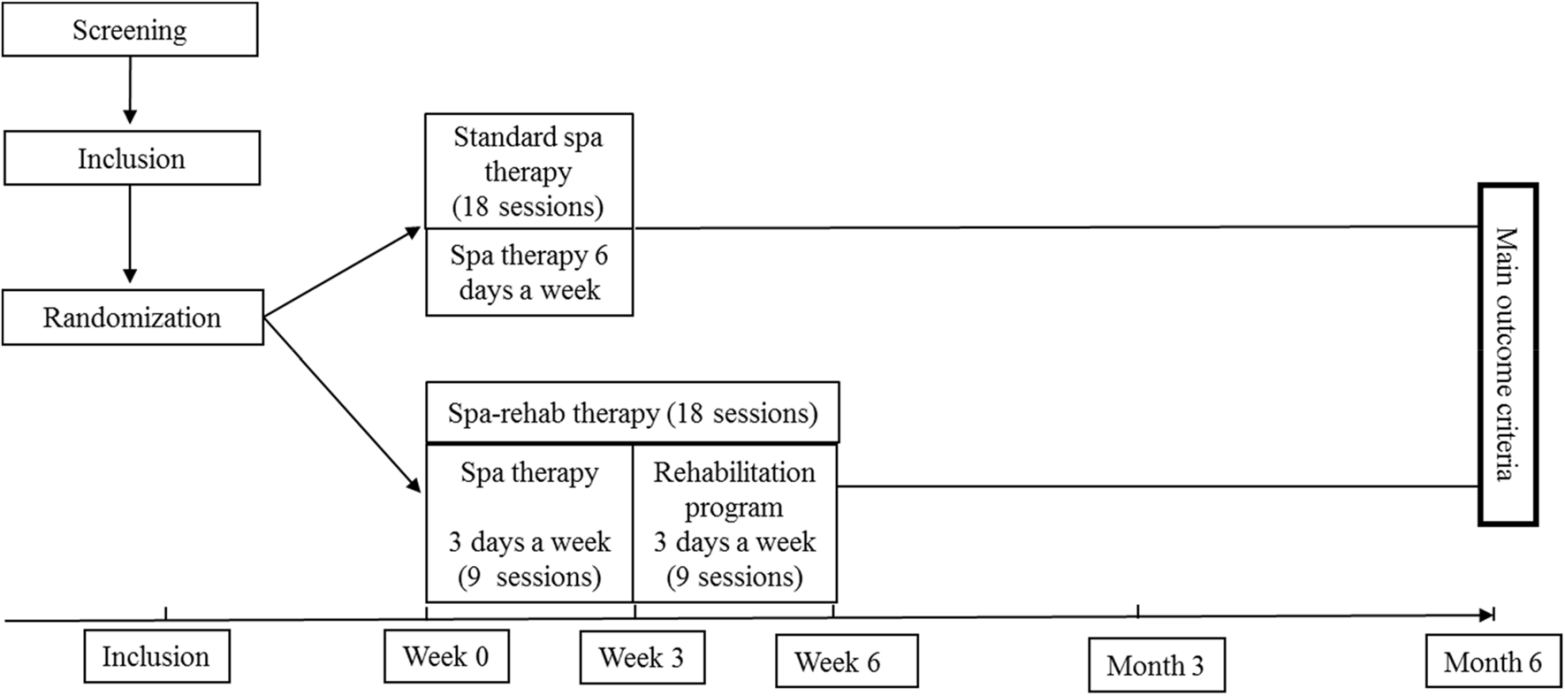
Design of the study.

We conducted a prospective, randomized, monocenter non-inferiority study comparing Spa-rehab and standard spa therapy for symptomatic KOA (Fig. 1).

### Intervention

#### Standard spa therapy

The standardized spa therapy protocol has been published and has been proven effective for KOA^12^. Patients received 18 days of spa therapy over 3 weeks, 6 days a week. Each day treatment included 4 techniques: mineral hydrojet sessions at 37 °C for 15 min, manual massages under mineral water at 38 °C by a physiotherapist for 10 min, applications of mineral-matured mud at 45 °C to the knee for 15 min and supervised general mobilization in a collective mineral water pool at 32 °C with groups of 6 patients for 25 min. The different sessions were interspersed with rest periods in a comfortable resting room.

#### Spa-rehab therapy (spa therapy + rehabilitation program)

Patients received 9 days of spa therapy over 3 weeks, for 3 days a week, with the same 4 techniques as for standard spa therapy. Then, patients followed a 9-day rehabilitation program over the next 3 weeks, for 3 sessions a week (supplemental material). This rehabilitation program aimed to maintain the effect of spa therapy; improve joint mobility, strength and proprioception; and develop appropriate behavior and attitudes. This program was designed by experienced rehabilitative physicians (J Paysant, R Ceconnello, P Boisseau) according to the Société Française de Chirurgie Orthopédique and Société Française de Médecine Physique recommendations^20,21^ and was personalized and adapted (type and intensity) for each patient. Rehabilitation sessions included 4 sequences: a preliminary sequence based on physiotherapy, personalized work with stretching exercises followed by muscular strengthening of the lower limb, global work with functional and proprioceptive exercises and a collective education session giving information on OA and treatment, and a program of exercises to perform at home. A booklet with all the information was delivered.

The Spa-rehab therapy was designed to improve the availability to follow spa therapy for patients with busy daily lives, to decrease fatigue perception and to extend the spa therapy effects.

All patients continued their usual non-steroidal anti-inflammatory drug (NSAID) or analgesic treatment.

### Patients

Inclusion criteria were meeting the American College of Rheumatology criteria for KOA^22^, unilateral or bilateral OA, pain on a visual analog scale (VAS, 0–100) > 30, and tibiofemoral Kellgren and Lawrence (K&L)^23^ grade ≥ 2. Exclusion criteria were total knee replacement surgery (TKR) scheduled in the next year, contraindication to spa therapy (evolving cardiovascular conditions, immune deficiency, cancer, current infection), severe comorbidity leading to significant deterioration of QoL, other symptomatic musculoskeletal disease, spa treatment within the previous 12 months, physiotherapy, rehabilitation or knee steroid injection within the past month and hyaluronic acid injection or change in symptomatic slow-acting drug use for OA in the past 3 months. Before the baseline visit, patients were asked not to change their NSAID regimen in the previous 5 days and their painkiller regimen in the previous 12 h.

Patients were recruited in the Lorraine region, east of France, by advertisements in the regional press and by posting flyers in waiting rooms of offices of general practitioners, rheumatologists, and physiotherapists. A free-access telephone number was available to take appointments. Patients were examined for inclusion criteria by rheumatologists working in the regional tertiary care center with no vested interest in the spa.

All patients gave their written informed consent before inclusion. The ethics committee “Comité de Protection des Personnes” (CPP) Est III gave approval for the study (2011-A01319-32; No. CPP: 11.12.02) and the study was registered at ClinicalTrials.gov (NCT01544647; March 6, 2012). The methods were carried out in accordance with the relevant guidelines and regulations. Spa mineral water and treatments were authorized by the French authority.

### Randomization

A computer program was used to randomly assign eligible patients to the 2 spa therapies. Because complete blinding of patients is not possible in non-pharmacologic treatments, the Zelen double-consent randomisation technique was used to avoid measurement bias and “resentful demoralisation” linked to not receiving the preferred treatment or another negative effect of preference bias: patients refusing to be included because they are not assigned to their preferred group^24^. Indeed, some patients could find the active spa therapy too long or not providing enough spa sessions, and some patients could find standard spa therapy too hectic and tiring, which could prevent them from participating because they have to stop their usual activities. Accordingly, patients were randomized and gave their signed inform consent after randomization^24,25^. Each treatment group had its own consent form and information letter explaining the practical details of the intervention. Also, to avoid measurement bias, treatment in the spa setting was not performed at the same time.

### Data collection

After the pre-inclusion visit and randomization, data were collected at baseline (beginning of spa therapy), at 3 weeks (end of spa therapy), 6 weeks (end of rehabilitation therapy for Spa-rehab group) and 3 and 6 months.

#### Outcome criteria

The primary endpoint was achieving at 6 months a minimal clinically important improvement (MCII), defined for KOA as ≥ 19.9 mm on a VAS for pain (0–100), and/or ≥ 9.1 points^26^ on the Western Ontario and McMaster Universities Osteoarthritis Index (WOMAC) function subscale (0–100)^27^ and no TKR – the “composite MCII”. Secondary endpoints were achieving at 3 and 6 months an MCII for pain and function and a patient acceptable symptom state (PASS) for pain (≤ 32.3 mm on a VAS for pain (0–100)), or function (≤ 31.0 on the WOMAC function subscale)^26^; evolution of pain and fatigue scores on a VAS (0–100); Medical Outcomes Survey 36-item Short Form (SF-36)^28,29^ and OsteoArthritis Knee and Hip Quality Of Life questionnaire (OAKHQOL) scores^30^; drug consumption; and satisfaction. We thus assessed the core set of patient-reported outcome measures (PROMs) (pain, physical function and patient global assessment) recommended^31^.

#### Other data collected

Sociodemographic data (age, gender, education level, marital status, employment status), and clinical data (comorbidity, body mass index, treatments, knee effusion, knee malalignment, previous injury, symptoms duration) were recorded at baseline. Knee X-rays, including a Schuss view of < 6 months, were graded according to the K&L classification^23^.

At each visit, the spa physician performed a clinical examination and inquired about drug consumption and adverse events (AEs).

Patients were asked to assess their level of pain and fatigue on a VAS (0–100) and to complete a functional ability survey [WOMAC (0–100): higher scores indicating greater severity]^27^ and QoL questionnaires [SF-36 and OAKHQOL (0–100): lower scores indicating greater severity]^29,30^. Patients were also questioned about their satisfaction with treatment on a VAS (0–100). The number of sessions each patient attended was recorded. Adherence to the program of exercises to perform at home for the Spa-rehab therapy group was not monitored.

Postural control was also measured by static posturography but reported elsewhere^32,33^.

### Safety analysis

All patients who attended at least one spa therapy session were included in the safety analysis. All AEs were reported whatever their severity. A serious AE was defined as any untoward medical occurrence that results in death, is life-threatening, requires inpatient hospitalization or prolongation of existing hospitalization or results in persistent significant disability or incapacity.

### Statistical analysis

Assuming a response rate of 50% with standard spa at 6 months^15^, a power of 80% and an alpha risk of 5%, we needed 135 patients per group to demonstrate the non-inferiority of Spa-rehab therapy as compared with standard spa therapy, with a lower confidence limit of the difference in proportions of 15%. To allow for an expected 5% attrition rate, we aimed to recruit 284 patients overall. Categorical data are expressed as number (%) and continuous data as mean ± SD. We checked differences between groups at baseline by Student *t* test and chi-square test as appropriate. Per-protocol analyses were performed to maximize the contrast between the 2 groups^34^ and intent-to-treat analysis to preserve randomization^35^. Patients who failed to attend half of the sessions were not included in the per-protocol analysis. As recommended in non-inferiority trials^34,36^, the 90% CI for the difference was used and the Spa-rehab therapy was considered not inferior to the standard spa therapy if the lower boundary of the 90%CI for the difference between the 2 groups (Spa-rehab minus standard spa therapy), was not < 15% for the primary endpoint (proportion of patients achieving the composite MCII). The same analyses were repeated at 3 months for the proportions of patients achieving the MCII for pain, MCII for function and composite MCII. The proportion of patients achieving the PASS for pain and function was computed before spa therapy and at 3 and 6 months after therapy and compared by the non-inferiority tests as above. The evolution of function and QoL are reported graphically. We computed the standardized ES, equal to the mean change in score from baseline to 6 months, divided by the SD of the baseline score in each group. The relationship between baseline variables (sociodemographic and clinical data, X-rays, season) and proportion of patients achieving the composite MCII was analyzed by logistic regression analysis. Candidate variables with *p* < 0.1 on bivariate analysis were included in the multivariate models. Variables with *p* < 0.05 were retained in the models. Between-group comparisons of AEs were analyzed by chi-square test. All analyses involved use of SAS 9.3 (SAS Inst., Cary, NC). *p* < 0.05 was considered statistically significant.

## Results

We screened 566 patients between February and September 2013; 305 were eligible, pre-included and randomized. Overall, 22 patients declined participation before the inclusion visit for logistic reasons (too-long delay between signing the consent form and the intervention or unable to find available time or transportation). Overall, 283 remaining participants were allocated to standard spa (n = 145) or Spa-rehab therapy (n = 138). None of the patients refused the treatment allocated when they signed the consent form.

**Figure 2:**
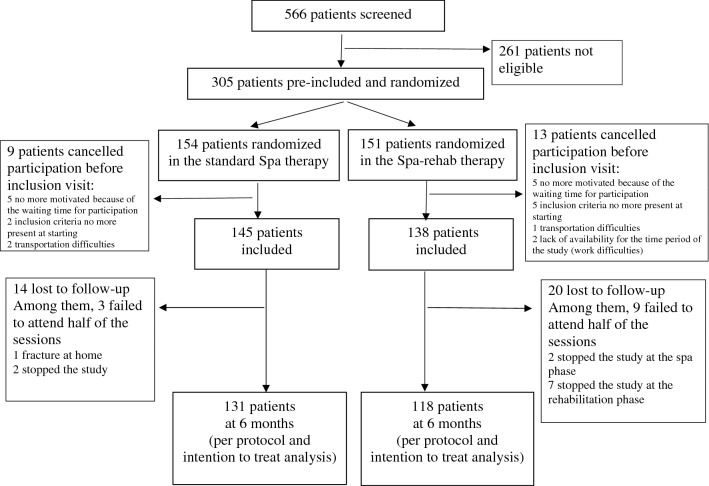
Study flow chart.

Twelve patients failed to attend half of the sessions and were not included in the per-protocol analysis, but they were also lost to follow-up, so the population for per-protocol and intent-to-treat analyses was the same. In all, 131 (92.3%) patients with standard spa therapy and 118 (91.5%) with Spa-rehab therapy completed the 6-month follow-up (Fig. 2).

Among the patients included in the analysis, the mean (SD) number of days of spa therapy completed was 17.7 (0.7) in the standard spa therapy group (18 were expected) and 8.7 (0.9) in the Spa-rehab therapy group (9 were expected). Over the 9 days of rehabilitation expected, the mean (SD) number of days completed was 8.7 (0.5).

### Patient characteristics

The baseline characteristics of patients are in Table 1. Baseline sociodemographic or disease characteristics of patients did not differ between the groups, except that pain and functional disability was greater with standard than Spa-rehab therapy (Table 1), but the differences were low: pain and functional disability difference of 4.1 and 5.0, respectively, on a 0–100 scale.

**Table 1:** Baseline characteristics of patients with knee osteoarthritis (OA) undergoing standard or Spa-rehab therapy.

	Standard spa therapy				Spa-rehab therapy				*p*
	N = 145				N = 138				
	N	(%)	Mean	(SD)	N	(%)	Mean	(SD)	
Age			64.9	(9.2)			63.2	(8.9)	0.10
**Gender**									
Women	105	(72.4)			85	(61.6)			0.05
**Education**									
Primary school	25	(17.6)			11	(8.0)			0.06
Secondary school	74	(52.1)			79	(57.7)			
University	43	(30.3)			47	(34.3)			
**Marital status**									
Married/partner	103	(71.5)			95	(68.8)			0.62
**Employment status**									
Employed	43	(29.7)			42	(30.4)			0.89
Symptom duration (years)			12.6	(10.4)			11.2	(11.2)	0.20
BMI (kg/m^2^)			29.2	(5.4)			29.1	(6.1)	0.61
Pain VAS (0–100)			53.6	(16.6)			49.5	(19.2)	0.04
WOMAC function (0–100)			43.8	(16.9)			38.8	(16.0)	0.01
Bilateral knee OA	97	(78.9)			93	(82.3)			0.51
Comorbidity Groll index [1–18]			2.7	(1.3)			2.4	(1.1)	0.07
Rupture of cruciate ligaments			11	(7.8)			4.0	(3.1)	0.10
**Maximal K/L grade of the TF joint**									0.57
Grade 2	58	(40.0)			59	(43.4)			
Grade 3	34	(23.4)			48	(35.3)			
Grade 4	53	(36.6)			29	(21.3)			
At least 1 painkiller (during the past month)	112	(77.2)			92	(66.7)			0.05
At least 1 NSAID (during the past month)	69	(47.9)			60	(43.5)			0.49

*BMI* body mass index, *VAS* visual analogue scale, *WOMAC* Western Ontario and McMaster Universities OA Index, *K/L* Kellgren and Lawrence system grades of OA, *TF* tibiofemoral, *NSIADs* non-steroidal anti-inflammatory drugs.

As well, patients lost to follow-up or not respecting the protocol (n = 34) and patients completing the study (n = 249) did not differ except for employment status: 17 (50%) were retired among those lost to follow-up and 182 (72.7%) in the completer group (*p* = 0.007).

One patient in each group underwent TKR between the 3- and 6-month visits.

### Primary outcome measure

At 6 months, the 90% CI for the difference in responders between the two groups according to the composite MCII ranged from − 0.18 to 0.02 (*p* = 0.14), which excluded the non-inferiority of the Spa-rehab therapy (Table 2) (− 0.18 was below the non-inferiority limit of 15%). Analysis was completed with chi-square test assessing the difference between the 2 groups, which was not significant at 6 months (*p* = 0.18). Adjustment on baseline VAS for pain did not change the result.

**Table 2:** Number of patients achieving the minimal clinically important improvement (MCII) at 6 and 3 months.

Definition of responder	Standard spa therapy		Spa-rehab therapy		Difference for responders (Δ: Spa-rehab therapy minus standard spa therapy)			
	N	(%)	N	(%)	Δ	[90%CI]	*p* (non-inferiority)	*p* (difference)
**6 months**	n = 130		n = 114					
Composite MCII	86	(66.2)	66	(57.9)	− 0.08	(− 0.18 to 0.02)	0.14	0.18
Pain MCII	50	(45.9)	37	(39.4)	− 0.07	(− 0.18 to 0.05)	0.11	0.35
Function MCII	69	(56.1)	56	(50.0)	− 0.06	(− 0.17 to 0.05)	0.09	0.35
**3 months**	n = 135		n = 107					
Composite MCII	76	(56.3)	75	(70.1)	0.14	(0.04 to 0.24)	< 0.0001	0.03
Pain MCII	49	(36.3)	49	(45.8)	0.10	(− 0.01 to 0.22)	0.0001	0.14
Function MCII	64	(47.4)	55	(51.4)	0.04	(− 0.07 to 0.15)	0.002	0.57

Composite MCII: achieving pain MCII defined for knee OA as ≥ 19.9 mm on a VAS pain scale (0–100), and/or function MCII defined for knee OA as ≥ 9.1 points on the WOMAC function subscale (0–100) and no knee surgery.

### Secondary outcome measure

At 3 months, the 90% CI for the difference between the two groups of responders according to the composite MCII ranged from 0.04 to 0.24 (*p* < 0.0001) (Table 2), which demonstrated the non-inferiority of the Spa-rehab versus standard spa therapy for the composite MCII. The 90% CI for the difference excluded 0, which indicates a significantly greater response with Spa-rehab than standard spa therapy.

The number of patients achieving the PASS increased from baseline to 3 months and then decreased at 6 months, especially with Spa-rehab therapy (Table 3). Non-inferiority was shown for all analyses (*p* = 0.007 to < 0.0001).

**Table 3:** Number of patients achieving the Patient Acceptable Symptom State (PASS) before spa therapy at 3 and 6 months.

Definition of responder	Standard spa therapy		Spa-rehab therapy		Difference for responders (Δ : Spa-rehab therapy minus standard spa therapy)		
	N	(%)	N	(%)	Δ	(90% CI)	*p* (non-inferiority)
**Before spa**	N = 145		N = 138				
PASS for pain	13	(9.6)	21	(16.2)	0.07	(− 0.001 to 0.13)	< 0.0001
PASS for function	32	(23.0)	40	(29.4)	0.06	(− 0.02 to 0.15)	< 0.0001
PASS for pain or function	35	(26.9)	44	(34.1)	0.07	(− 0.02 to 0.17)	< 0.0001
**3 months**	N = 136		N = 110				
PASS for pain	54	(44.6)	66	(66.0)	0.21	(0.11 to 0.32)	< 0.0001
PASS for function	62	(45.6)	73	(68.9)	0.23	(0.13 to 0.33)	< 0.0001
PASS for pain or function	73	(60.3)	82	(85.4)	0.25	(0.16 to 0.35)	< 0.0001
**6 months**	N = 131		N = 118				
PASS for pain	51	(44.0)	47	(45.6)	0.02	(− 0.09 to 0.13)	< 0.0001
PASS for function	63	(48.8)	72	(62.1)	0.13	(0.03 to 0.24)	< 0.0001
PASS for pain or function	72	(63.2)	74	(73.3)	0.10	(− 0.002 to 0.20)	0.007

**Figure 3:**
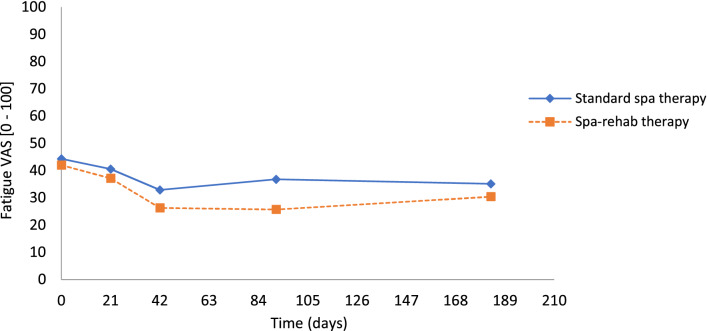
Evolution of fatigue over time in standard and in spa-rehab therapy groups measured on visual analog scale (VAS) at inclusion, 3 weeks, 6 weeks, 3 months and 6 months in both groups (blue points = standard spa therapy and orange squares = Spa-rehab therapy).

Pain and physical function dimensions of QoL or functional instruments improved at the end of spa treatment (3 weeks). They still improved from 3 to 6 weeks with Spa-rehab therapy and then reached a plateau up to 6 months. Emotional and vitality dimension scores also improved up to 6 weeks (supplemental material). Fatigue decreased with both therapies (Fig. 3).

The ES for the outcome measures between baseline and 6 months for the 2 groups are in Table 4.

**Table 4:** Effect sizes of the outcome measures between “before spa” and 6 months with the 2 spa therapies.

	Standard spa therapy	Spa-rehab therapy
	Effect size	Effect size
VAS pain [0–100]	1.06	0.91
VAS fatigue [0–100]	0.39	0.54
**WOMAC [0–100]**		
WOMAC function	0.76	0.75
WOMAC pain	0.93	0.98
**SF-36 [0–100]**		
Physical functioning	0.29	0.46
Physical role	0.61	0.51
Bodily pain	1.00	1.00
Mental health	0.40	0.31
Emotional role	0.27	0.33
Social functioning	0.45	0.48
Vitality	0.41	0.44
**OAKHQOL [0–100]**		
Physical activity	0.56	0.52
Pain	0.83	0.57
Mental health	0.27	0.29
Social activities	0.25	0.29

*VAS* visual analog scale, *WOMAC* Western Ontario and McMaster Universities Osteoarthritis Index, *SF-36* Medical Outcomes Survey 36-item Short Form, *OAKHQOL* OsteoArthritis Knee and Hip Quality Of Life questionnaire.

The number of patients who took at least one pain killer since the last visit decreased from baseline to 6 months: 72 (50.7%) to 36 (29.0%) with standard spa therapy and 81 (63.3%) to 43 (37.7%) with Spa-rehab therapy. Satisfaction on the VAS was greater with standard than Spa-rehab therapy (*p* = 0.01) at 3 weeks and was lower at 6 weeks (*p* = 0.01) (figures a, b, c, supplemental material).

### Factors associated with the spa therapy response

Factors independently associated with the number of responders according to the composite MCII were baseline WOMAC function score [OR for each 10-point increase, 1.8, 95% CI (1.5–2.2)] and the season of the 6-month visit [OR 2.7 (1.2–6.2); and 1.4 (0.7–3.1)], for completing the 6-month visit in autumn (n = 85) and winter (n = 88) versus spring (n = 53).

### Adverse events (AEs)

No serious AEs linked to the intervention were reported. Four serious AEs not linked to the intervention (ovarian cyst, uncontrolled diabetes, pulmonary embolism, hydrocele) were reported with standard spa therapy and 3 (prolapse, carotid surgery, TKR) with Spa-rehab therapy. These all occurred well after the spa treatment. The AEs are described in supplemental material (table d). Patients mainly reported knee or other musculoskeletal symptoms, fatigue, infections or cutaneous symptoms. The number of AEs did not differ with the 2 therapies (*p* < 0.2).

## Discussion

Non-inferiority of Spa-rehab versus standard spa therapy could not be demonstrated for the main outcome criteria (composite MCII) at 6 months. However, the difference test was not significant. Spa-rehab therapy was not inferior to standard spa therapy for the MCII criteria at 3 months or the PASS at 3 and 6 months.

Spa care or balneotherapy protocols are diverse, but few have demonstrated a clinically significant efficacy and they are rarely compared among themselves^37–39^. In a systematic review, only 9 RCTs analysing thermal mineral water care in KOA were included, with small sample sizes and short follow-up^9^. A Cochrane review of balneotherapy highlighted the poor quality of studies^40^. When present, the effect is usually small; however, the ES is comparable to that with medical treatment for OA^18^.

One large RCT of spa therapy showed a favorable effect on pain and function after 6 months as compared with usual treatment alone. However, the treatment was long and intensive (daily sessions during a 3-week period)^15^.

With our Spa-rehab therapy, designed to improve availability to follow spa therapy, to decrease fatigue perception and to prolong the spa therapy effects, pain and physical functioning dimensions improved at the end of the spa treatment and still improved from 3 to 6 weeks and then reached a plateau up to 6 months. Patients with Spa-rehab therapy showed better results at 6 weeks and 3 months than those with standard spa therapy, but more patients seemed to lose the clinically significant effect at 6 months. This finding confirms the short effect of rehabilitation^11^. Fatigue followed the same evolution and was similar in the 2 groups.

We found only 2 factors predicting the achievement of the MCII at 6 months: high WOMAC function score at baseline and season of assessment. The first factor can be explained in part by regression to the mean. The association with season of assessment could be explained by climatic conditions, changes in implementation of the spa protocol, longer delay between inclusion and treatment, and time of the year.

Some weaknesses of the study should be highlighted. First, despite randomization, pain and functional limitations in patients at baseline were slightly higher with standard spa than Spa-rehab therapy. This finding may have been responsible for a greater number of patients achieving the MCII with standard spa than Spa-rehab therapy and the lack of non-inferiority at 6 months^41^. Second, the results of the trial for the main criteria are difficult to interpret because we could not demonstrate non-inferiority or a difference between interventions. We calculated the sample size with an a priori hypothesis of an expected difference of 0, as is usual in a non-inferiority trial. If the hypothesis of the expected difference was 8%, as we observed, a post-hoc statistical power analysis showed that 600 subjects per group would have been required to demonstrate the non-inferiority, with 577 subjects per group to demonstrate a statistically significant difference from 0. However, a true difference of 8% between the 2 groups is not clinically significant, and we set the meaningful lower limit of non-inferiority at 0.15. The study was monocentric, which may limit the generalizability of the results. Adherence to the program of exercises to perform at home for the Spa-rehab group was not monitored. Finally, the objective of the study was to develop an intervention better suited to patients and easier to follow than the current standard spa protocol of 3 weeks and to demonstrate its non-inferiority but not its mechanism of action. Therefore, we did not investigate how and why the intervention works.

Our study has several strengths. First, PROMs are now widely used, because taking into account patients’ perception of their health state has become a priority. Analyzing the number of responders based on meaningful thresholds is an appropriate way to compare treatments but is rare in spa studies. As now recommended, data were analyzed considering a combination of change (e.g., MCII) and final state (e.g., PASS) to determine the response to the intervention^42^. Indeed, minimally important difference (MID)-only approaches tend to lack specificity. Few studies have included a number of patients sufficient to analyze the MID, and our study size was among the largest of studies of spa therapy in OA. Finally, the program of rehabilitation combining exercises to increase strength, flexibility and aerobic capacity with a large number of supervised sessions met actual recommendations^11^.

Future research is needed to determine the best protocol for maintaining work productivity in workers with symptomatic KOA and the intervention with the best cost-effectiveness ratio as compared with usual care. As well, we need to better understand how and why spa therapy is efficient^43,44^ because numerous elements play a role besides balneotherapy and physical exercises: remoteness from usual life environment, group dynamics, frequent contacts with health care professionals, absence of work duties and personal investment.

In conclusion, we could not demonstrate the non-inferiority of Spa-rehab versus standard spa therapy for the main outcome criteria at 6 months. However, the difference test was not significant. As well, Spa-rehab therapy was not inferior to standard spa therapy for the MCII criteria at 3 months or the PASS at 3 and 6 months. Spa-rehab therapy may be an acceptable alternative to standard spa therapy for patients with symptomatic KOA. This protocol could be cost-effective and allow patients who are still employed to benefit from spa therapy without absenteeism from work or for patients living close to the spa centre to avoid accommodation costs.

### Ethics approval and consent to participate

All patients gave their written informed consent before inclusion. The ethics committee CPP Est III gave approval for the study (2011-A01319-32; No. CPP: 11.12.02) and the study was registered at ClinicalTrials.gov (NCT015446476. March, 2012). Spa mineral water and treatments were authorized by the French authority.

## Supplementary information

Supplementary information

Supplementary information 2

Supplementary information 3

Supplementary information 4
