# Supplementary material for: Spa therapy with physical rehabilitation is an alternative to usual spa therapy protocol in symptomatic knee osteoarthritis
Source: Sci Rep. 2020 Jul 3;10:11004. doi: 10.1038/s41598-020-67436-1 (PMC7334225; doi:10.1038/s41598-020-67436-1)
Supplement: Supplementary file 1 — Supplementary information [file 41598_2020_67436_MOESM1_ESM.docx]

*Supplemental material*

**Spa therapy with physical rehabilitation is an alternative to usual spa therapy protocol in knee osteoarthritis**

Rat Anne-Christine*^1,2,3^, Loeuille Damien*^2,4^, Vallata Amandine^3,1^, Bernard Lorraine^3^, Spitz Emmanuel^2^, Desvignes Alexandra^2^, Boulange Michel^5^, Paysant Jean^6^, Guillemin Francis**^1, 3^, Chary-Valckenaere Isabelle**^2,4^

Annex 1: Spa-rehab therapy

Rehabilitation care will include the following:

1) preparatory work by the lower limbs based on local hot physiotherapy ("hot pac"), the participant being in a strict supine position with cushions under the head and under the popliteal cavity of the non-massaged knee, and manual massages with effleurage of the lower limb, sliding pressure or static pressure stepped from the root of the lower limb to the foot and back for deep and superficial venous system, maneuvers relaxing thigh, calf and foot muscles followed by circulating sliding pressures. This treatment can be completed with palpating-rolling in the peri-articular region of the knee and a global effleurage for a total duration of 20 min;

2) a personalized work consisting of musculo-tendinous stretching adapted to the initial assessment, carried out in stages or continuously, manually or by posture, followed by muscle strengthening, preferably static or in a chain, always adapted to the initial assessment and carried out against manual resistance, of the diagonal spiral Kabat type, or instrumental resistance, of the elastic, analytical or global resistance type;

3) global work, proprioceptive and functional, consisting of tests and eye balance exercises with eyes open and closed, on a Satel or Biorescue rehabilitation platform and on carpets and locomotion work on standardised walking paths;

4) a group educational session according to a predefined program with information about the disease, drug and non-drug OA management, risk factors (activities and weight), footwear and technical aids, lifestyle advice and exercises to be done at home and the hand-delivery of a follow-up booklet.

In the middle of the course, these sessions will be based on question-and-answer questionnaires, learning self-exercises and learning attitudes and gestures to be practiced in high-risk situations. At the end of the cure, these sessions will be conducted to self-correct exercises and gestures as well as the implementation of a physical activity program to be continued.

Physiotherapy care will be individualized and adapted (type and intensity of the exercises) to the initial physiotherapy assessment according to the recommendations of SOFCOT (Society for Orthopaedic Surgery) and SOFMER (Society of Medicine and Rehabilitation) from 2007.
