# Supplementary material for: Spa therapy with physical rehabilitation is an alternative to usual spa therapy protocol in symptomatic knee osteoarthritis
Source: Sci Rep. 2020 Jul 3;10:11004. doi: 10.1038/s41598-020-67436-1 (PMC7334225; doi:10.1038/s41598-020-67436-1)
Supplement: Supplementary file 2 — Supplementary information 2 [file 41598_2020_67436_MOESM2_ESM.pdf]

Spa therapy with physical rehabilitation is an alternative to usual spa therapy protocol in knee osteoarthritis

Rat Anne-Christine*[1,2,3], Loeuille Damien*[2,4], Vallata Amandine[3,1], Bernard Lorraine[3], Spitz Emmanuel[2], Desvignes Alexandra[2], Boulange Michel[5], Paysant Jean[6], Guillemin Francis**[1, 3], Chary-Valckenaere Isabelle**[2,4]

a. Evolution of pain and global satisfaction with standard spa and Spa-rehab therapy.

pain

global satisfaction

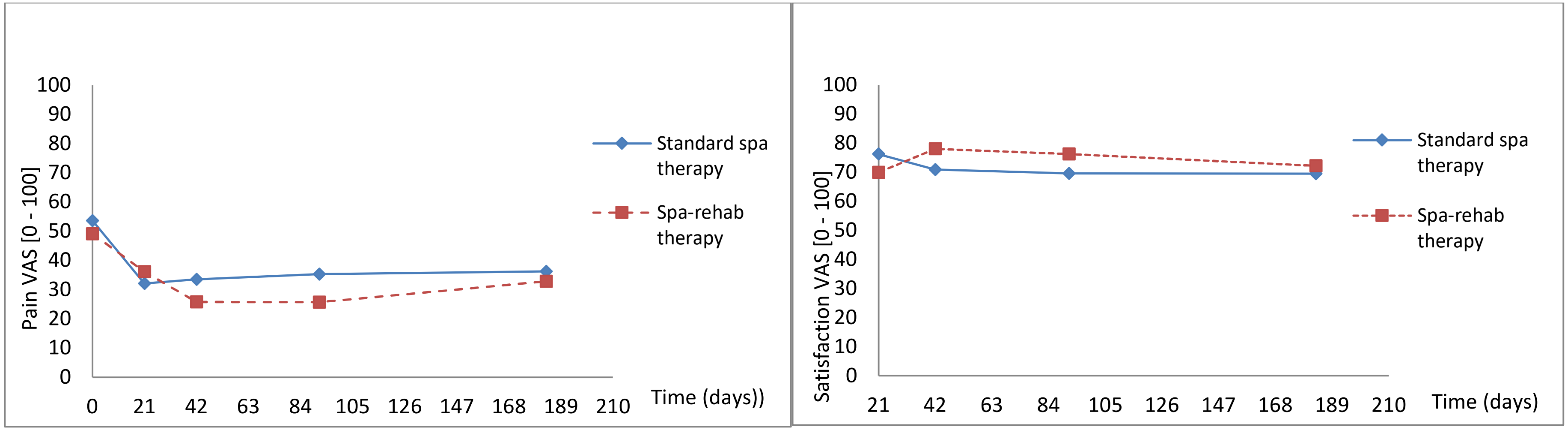

b. Evolution of WOMAC scores with standard spa and Spa-rehab therapy.

Standard spa therapy

Spa-rehab therapy

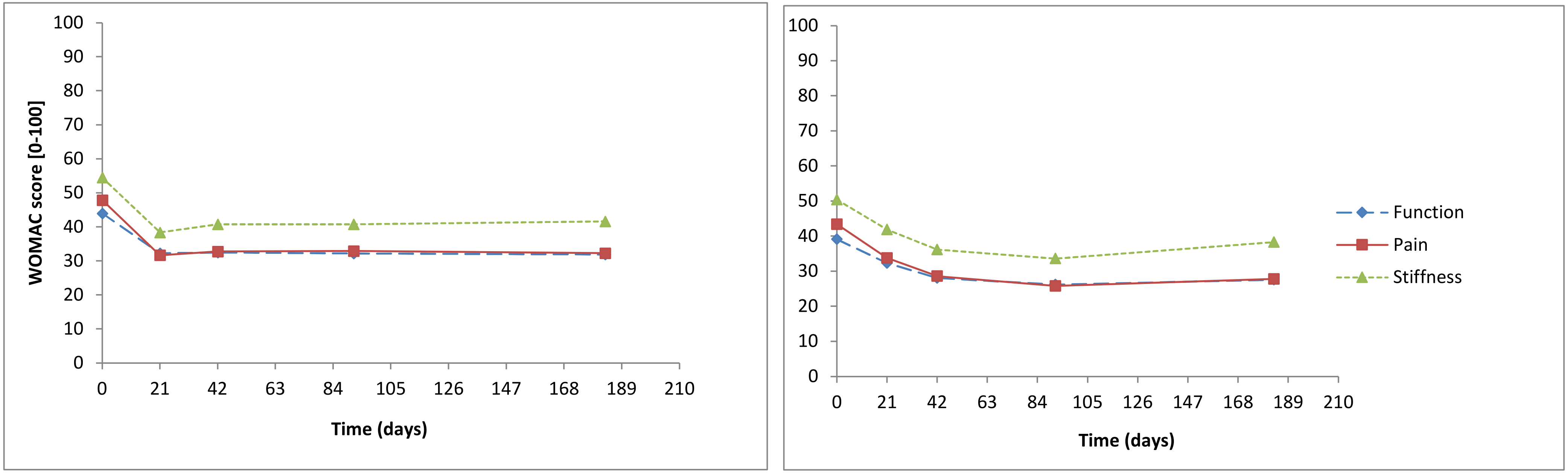

c. Evolution of QoL scores (SF36 and OAKHQOL) with standard spa and Spa-rehab therapy.

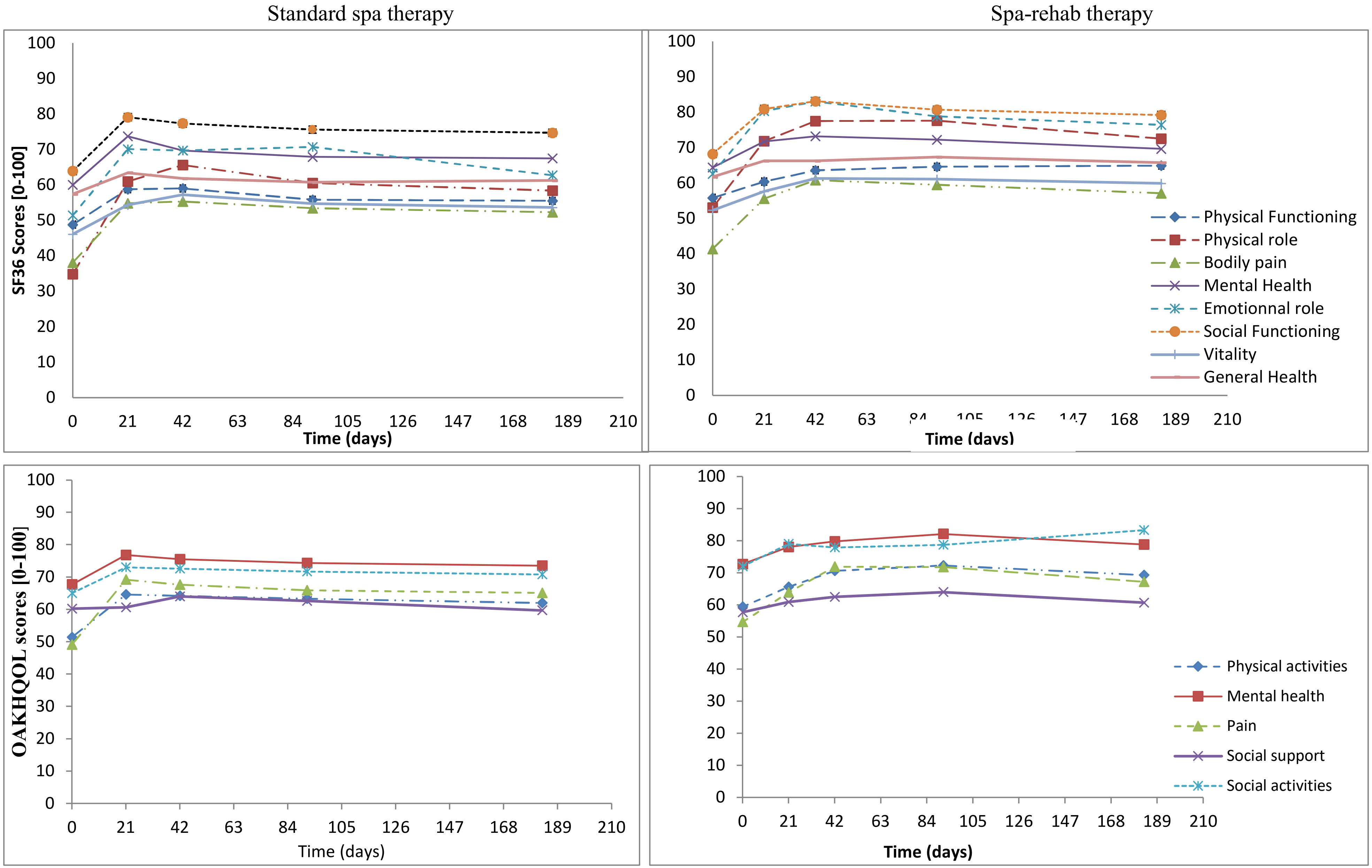

d. Adverse events with standard spa and Spa-rehab therapy.

Adverse events	Standard spa therapy N = 142		Spa-rehab therapy N = 126		Clinical manifestations
	N	%	N	%	
Musculoskeletal					
Knee	22	15,5	17	13,5	Exacerbation of pain, effusion
Other	17	12	16	12,7	Multifocal pains, back pain
Fatigue	27	19	11	8,7	
Cutaneous	23	16,2	8	6,3	Rash, pruritus, intertrigo, nails coloration
Infectious	9	6,3	3	2,4	Bronchitis, cystitis
Other	17	12	7	5,6	Hypertension, malaises, dizziness, fractures, falls
