# Supplementary material for: Spa therapy with physical rehabilitation is an alternative to usual spa therapy protocol in symptomatic knee osteoarthritis
Source: Sci Rep. 2020 Jul 3;10:11004. doi: 10.1038/s41598-020-67436-1 (PMC7334225; doi:10.1038/s41598-020-67436-1)
Supplement: Supplementary file 4 — Supplementary information 4 [file 41598_2020_67436_MOESM4_ESM.docx]

**Translated from French**

Nancy Thermal V1.2 20.10.2011

NANCY UNIVERSITY HOSPITAL CENTER - Research and Innovation Department

Clinical Research Protocol

Favourable opinion of the CPP East III on 09/02/2012 (N°: 2011-A01319-32 ; N° CPP : 11.12.02)

Authorization by the AFSSAPS on29/11/2011 (B111462-20)

Version N°1.2 of 20/10/2011

N°EUDRACT/ N° IDRCB : 2011-A01319-32

Clinical trials (N° : NCT01544647).

PROJECT TITLE:

COMPARISON OF TWO TYPES OF THERMAL MANAGEMENT IN SYMPTOMATIC KNEE OSTEOARTHRITIS: NANCY THERMAL STUDY RANDOMIZED MONOCENTRIC CLINICAL TRIAL

Sponsor :

Centre Hospitalier Universitaire de Nancy

Avenue du Maréchal de Lattre de Tassigny

54035 NANCY Cedex

Principal Investigator/Coordinator:

Name: Pr I. Chary-Valckenaere

Rheumatology, University Hospital Nancy

Tel: 03 83 83 15 32 03

Mail: [i.valckenaere@chu-nancy.fr](mailto:i.valckenaere@chu-nancy.fr)

Scientific manager:

Prof. D. Loeuille, Rheumatology, University Hospital Nancy

Methodological manager:

Dr AC Rat, Rheumatology and Centre for Clinical Epidemiology Inserm CIC-EC

Clinical Epidemiology and Evaluation Service

Nancy University Hospital

Scientific Committee:

Dr R Aussedat, Pr M Boulange, Mr Ceconnello, Pr I. Chary-Valckenaere, Pr D.Loeuille, Pr J Paysant, Pr Ph Perrin, Dr AC Rat, Dr V. Royaux, Dr S Ruscade-Cholay

Steering Committee:

Mr. Ceconnello, Pr I. Chary-Valckenaere, Pr F Guillemin, Pr D. Loeuille, Pr J Paysant, Dr. AC Rat, Dr. V. Royaux, Dr. S Ruscade-Cholay

Oversight Committee:

Pr P Gillet, Pr F Tubach, Pr X Chevalier, Pr C. Tavernier

Methodological and logistical support:

Pr F Guillemin Centre d'Epidémiologie Clinique Inserm CIC-EC

Clinical Epidemiology and Evaluation Service

Nancy University Hospital

Funder:

Urban Community of Greater Nancy

Represented by JY Folzer (DGA Pôle Services Urbains CUGN)

Investigating physicians responsible for patient inclusions: Dr. R Aussedat, Pr I. Chary-Valckenaere, Prof. D. Loeuille, Dr AC Rat, Dr E Spitz.

Investigating doctors in charge of thermal care: under recruitment

Research locations:

- patient inclusion: rheumatology department CHU Brabois

- thermal treatments: Nancy Thermal

Head of research acting on behalf of the promoter / and authorised to sign the Protocol and any amendments thereto on behalf of the sponsor:

Research and Innovation Department

Ph. BOULANGE - Director of Research and Innovation

Saint Julien Hospital - Rue Foller

54035 NANCY Cedex

SUMMARY

1. SUMMARY OF THE PROTOCOL

2. SCIENTIFIC RATIONALE AND GENERAL DESCRIPTION OF THE RESEARCH

3. RESEARCH OBJECTIVES

4. RESEARCH DESIGN

5. SELECTION AND EXCLUSION OF PEOPLE FROM RESEARCH:

6. TREATMENT / MEDICAL DEVICE

7. EFFECTIVENESS EVALUATION

8. SAFETY ASSESSMENT

9. STATISTICS

10. RIGHT OF ACCESS TO SOURCE DATA AND DOCUMENTS.

11. QUALITY CONTROL AND ASSURANCE.

12. ETHICAL AND REGULATORY CONSIDERATIONS

13. DATA PROCESSING AND DOCUMENT STORAGE AND RESEARCH DATA.

14. FINANCING

15. RULES RELATING TO PUBLICATION.

16. LIST OF APPENDICES

17. BIBLIOGRAPHICAL REFERENCES

| Title | Comparison of two types of thermal management in symptomatic knee osteoarthritis: Nancy Thermal study  Randomized monocentric clinical trial |
| --- | --- |
| Sponsor | Nancy University hospital |
| Coordinating investigator | Pr I. Chary-Valckenaere, Rheumatology department, Nancy University hospital |
| protocole | V1.2 20 October 2011 |
| center(s) | Nancy |
| Research | Biomedical research |
| BACKGROUND | Osteoarthritis (OA) is the most common rheumatological condition. Its distribution is universal. It is a public health issue because its socioeconomic consequences are considerable.  Physical treatments (spa treatment, muscle strengthening, etc.) are part of the recommendations for the therapeutic management of knee OA (EULAR, ACR, and the most recent recommendations, OARSI in 2010), but we lack a standardised protocol to optimise such treatments in clinical practice. In a recent publication, Forestier et al. showed, in a randomized multicentre study, "Thermarthrosis", the symptomatic efficacy of a standardized spa treatment protocol in knee OA on pain and function at 6 months. This standardized spa therapy includes 4 spa treatments: 1) hydrojets (individual), 2) massage/physiotherapy (individual), 3) mud applications (individual) and 4) balneotherapy (collective) repeated 6 days a week for 3 weeks. The results of this study show a beneficial effect of the treatment evaluated globally. This therapeutic action, whose philosophy is based on treatments carried out in discharge for a supporting joint, essentially involves their local sedative and anti-inflammatory action.  However, this type of care is sometimes considered tiring for patients who complain about its intensity and the difficulties of availability. Outpatient care, less intensive at a rate of 3 instead of 6 treatments per week but over a longer period of time combining standardised treatment and functional re-education, could be an interesting alternative to the current conventional treatment. After a sedative phase, it seems logical to recondition the patient for exercise by correcting the proprioceptive disorders and underlying muscle deficiencies, to prolong the long-term effectiveness of the spa treatment.  However, no study has evaluated the interest of combining a so-called sedative phase provided by spa therapy followed by an active approach of muscle strengthening and proprioception in knee OA. Moreover, no study has evaluated the factors predicting a good response to spa treatment. |
| MAIN OBJECTIVE | The main objective of the study is to compare the proportion of patients who achieved a composite outcome criteria combining the Minimal Clinically Important Improvement (MCII) in pain and/or functional ability and surgery of the target knee at 6 months with two types of spa therapy ("classical spa therapy" and "active spa therapy") in symptomatic knee OA. |
| Secondary objectives | The secondary objectives are to 1) compare the effectiveness of the two types of spa therapy on functional abilities and pain at the end of the treatment and at 3 months; 2) determine the evolution of quality of life scores (OAKHQOL, SF-36), fatigue, patient satisfaction, consumption of drugs and care, as well as the use of surgery in both types of spa therapy; 3) describe the effectiveness of the "classical spa therapy" in Nancy Thermal on pain and functional abilities by analyzing the before-and-after effect; 4) describe the postural anomalies encountered in patients with knee OA by posturographic analysis and evaluate the evolution of these anomalies according to the type of spa therapy over time; and 5) study the predictive response factors at 3 and 6 months |
| Main outcome criteria | The Minimal clinically important improvement (MCII) in OA was determined by F. Tubach et al. The MCII is a threshold for improvement that is particularly suitable for therapeutic trials and takes into account that the Minimal Clinically Important Difference (MCID) may be different for an improvement or a worsening. A patient is considered improved if the WOMAC index function subscale score is improved by at least 9.1 points from the initial value and/or if the current pain measured on a visual analog scale (VAS, 0-100 mm) is improved by at least 19.9 mm. These values correspond to the smallest clinically relevant change for these two outcome criteria. Improvements below this value will be considered as treatment failures. Patients with improvement but who would have still need to have surgery during the period of study will also be considered failures. The effect of two types of spa therapy ("a standard spa therapy" and an "active spa therapy") for symptomatic knee OA will be assessed by the proportion of patients who have achieved a composite criterion combining minimal clinically important improvement (MCII) for pain and/or functional abilities and the absence of knee surgery of the target knee at 6 months. |
| secondary outcome criteria | Secondary criteria   - Percentage of patients reaching MCII (pain, function and composite score) at the end of the treatment and at 3 months - Percentage of patients reaching the Patient Acceptable Symptom State (PASS) (pain and function) at 3 and 6 months. - Evolution of quality of life scores (OAKHQOL, SF-36) at 3 and 6 months - Evolution of VAS-fatigue, VAS-pain at 3 and 6 months - Evolution of WOMAC scores (function) at 3 and 6 months - Patient satisfaction (overall VAS or Health Education Impact Questionnaire **(**heiQ) module 9) at 3 and 6 months - Evolution of drug and care consumption, use of surgery - 6-minute walking test [43] - Evolution of posturography parameters: modification of the length travelled and/or the area covered by the pressure centre of the feet following the removal or disruption of sensory inputs, somesthesics of balance in distorted visual situations and static and dynamic positions [4] - adverse reactions |
| METHODOLOGY/ STUDY DESIGN | This is a single-centre, randomized, single-blind clinical trial of non-inferiority comparing "standard spa therapy" and "active spa therapy" in knee OA.  The patients in the standard spa therapy group will undergo a “classical spa therapy” according to the standardised care protocol defined in Thermarthrosis [1], that is, 1 daily session, 6 days a week for 3 weeks. The patients in the active spa therapy group will undergo therapy comprising thermal treatments of the classical spa therapy at a rate of 3 sessions per week for 3 weeks, followed by proprioceptive re-education and muscle strengthening at a rate of 3 sessions per week for 3 weeks. In both groups, patients will be allowed to take any additional medication and care that their referring physician deems necessary.  Randomization will be blinded and will use the Zelen method (randomization before consent is obtained).  Patients will be examined at 3 and 6 months by an evaluating physician, independent of the spa, who will be blinded to the patient's treatment group.  As a non-inferiority trial, the analysis will be per-protocol. |
| SUBJECT INCLUSION CRITERIA | Patients with knee OA:   - Meeting the criteria for knee OA of the American College of Rheumatology (*) - Uni- or bilateral OA - Symptomatic: VAS--pain > 3 on a scale of 0 to 10 - Radiological femoro-tibial knee OA³ stage 2 of the Kellgren and Lawrence classification - Providing consent - Preliminary medical examination   * The diagnostic criteria used are those of the ACR [Altman 1986]:   - Knee pain and at least 1 of the following 3 criteria:   - Age > 50 years old   - Morning stiffness < 30 min   - Crepitus on active motion - Presence of osteophytes on knee X-rays   (sensitivity 94%, specificity 87%) |
| CRITERIA FOR NON-INCLUSION | - Isolated patellofemoral OA - Indication for total joint replacement surgery within 1 year - Contraindication to spa therapy (immune deficiency, moderate to severe heart failure, cancer, infection, skin wound etc.) or predictable intolerance to spa therapy (intolerance to heat, baths, hot tubs, swimming pool etc.) - Other disease of the musculoskeletal system (inflammatory arthritis, disabling lumbar radiculopathy, motor deficit of the lower limbs) - Severe comorbidities - Severe depressive syndrome, psychosis - Previous knee treatments:   - Massages, physiotherapy, acupuncture of < 1 month   - non-steroidal anti-inflammatory drugs (NSAID) change within the last 5 days   - Change of analgesics within the last 24 hr   - Change of symptomatic slow-acting drugs in osteoarthritis of less than 3 months.   - Intra-articular injection of the target joint of < 1 month for corticoids and < 3 months for viscosupplementation   - Physiotherapy or rehabilitation of < 1 month.   - Spa therapy during the previous year |
| METHODS OF RECRUITING SUBJECTS | Information on the Nancy Thermal study will be provided to general practitioners and rheumatologists (mail and/or information meeting) in the months preceding the start of the study. Patient information will be provided through the press (regional press release and local radio stations) and by a poster campaign in the offices of physiotherapists, pharmacies, biological laboratories, and the waiting rooms of general practitioners and rheumatologists. A call centre will be set up to plan appointments with investigating physicians. |
| NUMBER OF SUBJECTS REQUIRED (PATIENTS, CONTROLS IF ANY) | Number of subjects required   - Main objective: with 50% responders to the standard spa therapy at 6 months [1], a power of 80%, and an alpha risk of 5%, 135 patients per group are required to significantly demonstrate the non-inferiority of the active spa therapy with a lower confidence limit of the difference in proportions of 15%. Considering that the number of patients lost to follow-up may be 5%, a total of 284 patients is needed in the trial. |
| LENGTH  DURATION | Expected inclusion period: January 2012 to the end of October 2012, i.e., 10 months  Duration of participation (including follow-up period): for a patient, 6 months from the beginning of the treatment  Total duration (including data analysis time): from December 2011 to mid-October 2013, i.e., 2 years |
| INVESTIGATION PROCEDURE DIFFERENCES FROM USUAL CARE | Spa therapy is performed according to the usual scheme for the "classical spa therapy”. The "active spa therapy” follows a pattern with fewer thermal care sessions, which are replaced by functional rehabilitation sessions by physiotherapists according to usual techniques.  The Nancy Thermal site does not yet have thermal approval and this study is carried out with this objective in mind. The chemical qualities of sodium chloride-containing water are identical to those of the Amnéville les Thermes spa.  Visits are made at inclusion, at the beginning of the cure, at 3 and 6 weeks and at 3 and 6 months. |
| EVALUATION OF RESEARCH BENEFITS AND RISKS | - The expected benefits are a reduction in pain, functional discomfort, and drug use and an improvement in quality of life.  - Adverse reactions that can be experienced with thermal treatment are rare, most often minor and transient. They are traditionally observed during the thermal treatment period and are then part of what is usually called the "thermal crisis": it is mainly a question of fatigue, awakening of certain pains during the thermal treatment phase, unusual sweating and insomnia. These effects rarely require the cessation of thermal treatments.  More serious health problems are possible with thermal treatment but remain exceptional: heart problems, infections linked to bacteria that may be present in warm waters (among them Legionella infection). |
| STATISTICAL ANALYSIS | **Descriptive statistics**  **Comparison of groups**  The trial being a non-inferiority trial, the main analysis will be a per-protocol analysis to be conservative by increasing the contrast between the groups.  Main objective  The active spa therapy will not be considered inferior to the standard spa therapy if the lower bound of the confidence interval at 90% of the difference between the 2 groups (active spa therapy minus standard spa therapy) for the main outcome criterion (percentage of respondents according to the composite criterion) is not less than 15%. After verification of the randomization that could be unbalanced due to the Zelen randomization mode, the final analysis will be adjusted if necessary on the variables that differ between the 2 groups.  Secondary objectives  - The same analyses will be repeated for the MCII composite criterion, pain and function at 3 months, and PASS criteria.  - To determine the predictive factors of spa therapy response, a bivariate analysis will be performed using chi-square tests (or Fisher exact tests depending on the number) for discrete variables and Student t-tests, ANOVA or Wilcoxon tests for quantitative variables. The parameters significant on bivariate analyses (at the conservative threshold of p=0.1) will then be introduced into a stepwise multiple logistic regression.  P<0.05 will be considered statistically significant.  The statistical analysis will be carried out by the Centre for Clinical Epidemiology by using SAS v9.2 (SAS Inc., Cary, NC). |
| EXPECTED FALLBACK | To be able to offer different types of spa therapy in order to adapt to patients' preferences.  Propose a spa therapy that can be performed externally near the patient’s home.  Offer a less tiring cure.  Obtaining thermal approval for the Nancy Thermal site. |

2. SCIENTIFIC RATIONALE AND GENERAL DESCRIPTION OF THE RESEARCH

Knee osteoarthritis (OA) is a common disease, the prevalence of which is increasing with age. Thus, an estimated 40% of the population over 75 years of age has OA [6]. In France, this represents 7.3% of the reasons for consultation in rheumatology, ahead of low back pain (6.1%) [7].

In addition to the important daily functional impact on patients, OA accounts for a considerable proportion of health expenditure, with direct costs estimated at €1.6 billion in France in 2002 [7]. These expenses are growing exponentially (+156% compared to 1993), due in particular to the ageing of the population and an increase in life expectanc, but also an increase in cost for patient care. Knee OA represents the third site in terms of frequency after lumbar OA and hand OA. Its painful and functional impact is important and the origin of pain is multifactorial: bone, inflammatory (effusion) and ligamentary [8].

Currently, knee OA is defined as a local disease characterized by destruction of joint cartilage and damage to other joint structures (bone, meniscus, synovium) and abarticular aspects (ligaments) even if some systemic factors may play a role. This disease may be aggravated by systemic factors (adipocytokines, growth factors, pro-inflammatory cytokines), vascular factors (arteriosclerosis of subchondral bone feeder vessels) and neurological factors (failure of the proprioceptive system) [9,10,11].

The diagnosis of OA is based on both clinical and radiographic features (joint space narrowing and bone reaction [osteosclerosis, osteophytes, geodes]) [5]. Biologically, we lack systemic inflammation and cartilage biomarkers for clinical follow-up or to judge the effectiveness of a treatment. Knee OA evolves for more than a decade, and the factors associated with clinical worsening and joint destruction are now better known and allow for better clinical evaluation of patients and thus a better optimization of research for therapeutic purposes [12]. Some risk factors are strongly associated with the aggravation of knee OA (malalignment, generalized OA) and others are moderately associated (intensity of symptoms at the beginning of the disease, duration of disease progression, synovial inflammation, ultrasonography and MRI findings, and hyper T2 signals of the trabecular bone on MRI) [8].

Therapeutic management of OA and in particular knee OA involves equal parts pharmacological and non-pharmacological treatment [13]. These treatments have effects on both pain and function and can be assessed by using algo-functional indices or quality of life scores. Therapeutic response criteria were determined and are commonly used (Minimal Clinically Important Improvement [MCII], Patient Acceptable Symptom State [PASS], OA Research Society International [OARSI] criteria) [2,14,15].

**Recommendations for the therapeutic management of knee OA**

A number of literature reviews and recommendations for practice are available for knee OA. These recommendations are regularly updated according to research progress: recommendations of the EUropean League Against Rheumatism (EULAR) [17], the American College of Rheumatology (ACR) [18] and OARSI in 2010 [13]. The Society of Medicine Physics and Rehabilitation (SOFMER) also made recommendations in association with the French Society of Arthroscopy (SFA)[16]. Although these recommendations depend on the methodological quality of the published studies, which are sometimes open to criticism, they nevertheless remain a reference and are widely distributed.

The analysis of the literature shows a modest effect of pharmacological treatments (paracetamol, NSAIDs, symptomatic slow-acting drugs in OA) [13]. Non-pharmacological treatments, including muscle strengthening, aerobic exercise and physical care seem to bring the same relief as pharmacological treatments but with a lower level of evidence owing to the complexity of the interventions and the methodology used (no long-term study, difficulties of double-blinding, etc.) [13]. Although the physical treatments (thermal cure, muscle strengthening etc.) are part of these recommendations, the interventions are not standardized, and the superiority of a specific technique or program compared to another has not been clearly established [13].

**Evaluation of crenotherapy**

Various good-quality thermal therapeutic trials have led to the recognition of spa therapy for treating chronic low back pain [19,20]. Several prospective randomized controlled trials have evaluated the effect of spa therapy for the main indications for crenotherapy in rheumatology: low back pain chronic, hip OA, hand OA, fibromyalgia, rheumatoid arthritis, psoriatic arthritis, spondylitis, chronic neck pain [21–35].

A number of these trials concern, at least in part, knee OA [36–42]. The estimated size effect of the treatment is 0.42, which corresponds to a moderate effect. [36, 39, 40]. In a recent article, Forestier showed in a randomized multicenter study, "Thermarthrosis", the efficacy of a standardized spa treatment protocol in knee OA on pain and function at 6 months [1]. This standardized cure involves 4 thermal treatments (hydrojets, massages, applications of mineral-matured mud at 45°C, balneotherapy) repeated 6 days a week for 3 weeks. The authors showed a symptomatic effect that remained evaluated globally and not according to its different components. This intervention, whose philosophy is based on care at discharge for a load-bearing joint also involves local sedative and anti-inflammatory care.

Although symptomatic efficacy may seem interesting, patients are more likely to sometimes complain about the intensity of this type of care, the fatigue and the difficulties with availability. External care, less intensive at a rate of 3 instead of 6 sessions per week but over a longer period of time, combining standardized spa therapy and rehabilitation, could be an interesting alternative to current conventional treatment. This approach combines the "sedative" action of spa therapy with muscular and proprioceptive strengthening. Indeed, after a sedative phase, it seems logical to recondition the patient for exercise by correcting the proprioceptive disorders and underlying muscle deficiencies, in order to prolong the long-term effectiveness of the spa treatment. However, no study has assessed the value of combining these two therapeutic approaches in knee OA.

**3. RESEARCH OBJECTIVES**

The purpose of this study is to compare the 6-month efficacy of two types of spa therapy: "standard spa therapy" (spa therapy delivered 6 days out of 7 for 3 consecutive weeks) and "active spa therapy” successively combining iterative spa sessions (3 sessions per week for 3 weeks) then specific re-education (3 sessions per week for 3 weeks).

The main objective of the study is to compare the proportion of patients with a composite response criterion combining the MCII for pain and/or functional ability and the absence of knee surgery on the targeted knee at 6 months, according to two types of spa therapy ("standard spa therapy" and "active spa therapy") in symptomatic knee OA.

The secondary objectives are as follows:

- To compare the effectiveness of the two types of spa therapy on pain and function at the end of the spa therapy and at 3 months

- To determine the evolution of quality of life scores (OAKHQOL, SF-36), of fatigue, patient satisfaction, medication use and care, as well as the use of surgery in both types of spa therapy

- To describe the effectiveness of standard spa therapy in Nancy Thermal on pain and functional abilities by an analysis of the before and after effect

- To describe the postural anomalies encountered with knee OA by a posturographic study and to evaluate the evolution of these anomalies according to the type of spa therapy and over time.

- To study the predictive factors of response to spa therapy (socio-demographic data, clinical, radiographic, ultrasonography and posturographic) at 3 and 6 months.

**4. STUDY DESIGN**

This is a single-centre, randomized, single-blind clinical trial of non-inferiority comparing "standard spa therapy" and "active spa therapy" in knee OA.

**3 weeks**

**6 weeks**

**M0**

**M3**

**M6**

**Inclusion**

**Randomization**

**standard spa therapy**

**Active spa therapy**

**End**

**d’étude**

Figure 1: Study design

**4.1. A PRECISE STATEMENT OF THE MAIN OUTCOME CRITERIA AND, WHERE APPLICABLE, SECONDARY OUTCOME CRITERIA**

Main Criterion

# The MCII in OA was determined by Tubach et al. [2]. The MCII is a threshold for improvement that is particularly suitable for therapeutic trials and takes into account that the MCID may be different for an improvement or a worsening. A patient is considered improved if the WOMAC index function subscale score is improved by at least 9.1 from the initial value and/or if the current pain measured on a VAS is improved by at least 19.9 mm. These values correspond to the smallest clinically relevant change for these two outcome criteria [2]. Improvements below this value will be considered treatment failures. Patients with improvement but would still need to have surgery during the study period will also be considered failures. The effect of the two types of spa therapy ("standard spa therapy" and "active spa therapy") for symptomatic knee OA will be assessed by proportion of patients who achieve a composite criterion combining the MCII for pain and/or functional abilities and the absence of knee surgery of the target knee at 6 months.

Secondary criteria

- Percentage of patients reaching the MCII (pain, function and composite score) at the end of the treatment and at 3 months
- Percentage of patients reaching the PASS (pain and function) at 3 and 6 months [14]. The PASS is the value beyond which the patient considers himself/herself to be well. It is defined as for the MCII by the 75th percentile of the score among patients whose assessment of the acceptable symptom state was “good” on a Likert scale with 5 answer modalities. The PASS for WOMAC function is 31 mm and for VAS for pain is 32 mm [14].
- Evolution of quality of life scores (Osteoarthritis Knee and hip Quality of life [OAKHQOL], SF-36) at 3 and 6 months
- Evolution of VAS-fatigue, VAS-pain at 3 and 6 months
- Evolution of WOMAC scores (function) at 3 and 6 months
- Patient satisfaction (overall VAS or heiQ module 9) at 3 and 6 months
- Evolution of drug and care consumption, use of surgery
- 6-minute walking test [43]
- Evolution of posturography parameters: modification of the length travelled and/or the area covered by the pressure centre of the feet after removal or disruption of sensory inputs, somesthesics of balance in distorted visual situations and static and dynamic positions [4]
- Adverse reactions

**4.2. DESCRIPTION OF THE RESEARCH METHODOLOGY, ACCOMPANIED BY ITS SCHEMATIC PRESENTATION SPECIFYING IN PARTICULAR THE VISITS AND EXAMINATIONS PREVUED.**

Collected data

Inclusion visit:

- Demographics: age, sex, level of education, current or former professional activity, place of residence (size of the municipality)
- Medical history and co-morbidities (Groll functional index)
- Medication
- History of knee OA: initial trauma (rupture of the cruciate ligament knee or lower limb fracture), presence of the symptoms before the current painful episode, duration of the current episode, previous drug treatments (corticosteroid injections, viscosupplementation, symptomatic treatments [analgesics, NSAIDs, symptomatic slow-acting drugs for osteoarthritis" (SySADOA)]) and non-drugs (surgery [osteotomy, arthroscopy, meniscectomy], spa treatment, rehabilitation, other etc.)
- Clinical data: weight, height, static disorders, length inequality of the lower limbs.
- X-rays of < 6 months with weight-bearing, full extended-knee X-ray and Schuss. The radiographs will be digitized for a centralized reading and will be used to confirm the diagnosis of femorotibial OA (Kellgren and Lawrence stage ≥2).
- Posturography examination
- Ultrasound examination

During other visits

Clinical and perceived health data will be collected before the cure (V0), at 3 weeks (V1: end of the standard spa therapy or change of type of care for active spa therapy), at 6 weeks (V2: end of active spa therapy), at 3 months (V3: M3) and 6 months (V4: M6) and will include the following examinations: clinical exam (effusion, joint amplitudes), VAS-pain, VAS-fatigue, WOMAC, OAKHQOL, SF-36, satisfaction, drug consumption, surgery, side effects, posturography examination, 6-minute walk. The number of treatments actually performed by the patients will also be collected.

The assessor will be blinded to the treatment group at 3 and 6 months.

**Table: Follow-up**

|  | **Selection inclusion** | **V0** | **V1**  **3** weeks | **V2**  **6** weeks | **V3**  **3 months** | **V4**  **6 months** |
| --- | --- | --- | --- | --- | --- | --- |
| Inclusion  Zelen randomization  Consent | X  X  X |  |  |  |  |  |
|  |  |  |  |  |  |  |
|  |  |  |  |  |  |  |
| Sociodemographic data | X |  |  |  |  |  |
| History of OA | X |  |  |  |  |  |
| Clinical examination | X |  |  |  |  |  |
| X-ray | X |  |  |  |  |  |
| Ultrasonography | X |  |  |  |  |  |
| Treatment | X | X | X | X | X | X |
| VAS pain | X | X | X | X | X | X |
| Clinical follow-up |  | X | X | X | X | X |
| WOMAC |  | X | X | X | X | X |
| Quality of life |  | X | X | X | X | X |
| Posturography |  | X | X | X | X | X |
| 6-minute walking test |  | X | X | X | X | X |
| Care consumption |  | X | X | X | X | X |
| Adverse reactions |  |  | X | X | X | X |
| Satisfaction, heiQ |  |  | X | X | X | X |

**4.3. DESCRIPTION OF THE MEASURES TAKEN TO REDUCE AND AVOID BIAS**

Randomization

Most literature reviews emphasize the importance of blinding of the assessor [44,45,46,47] and randomization [48,49,50] in a therapeutic trial. In the context of thermal care, it is impossible to blind the patient, which can lead to a bias of therapeutic preference [51]. The Zelen randomization method (randomization method before consent is obtained) must allow for reducing this bias. This method has been used successfully in the past [52,53]. For some, it has the additional advantage of reducing the number of refusals to enter the study [54]. A review published in 2006 shows that among 58 studies, this method was more frequently used to limit bias than to increase recruitment [55]. This method does not contradict the rules of ethics and is widely used in many studies.

Because patients cannot be completely blinded, randomization will be according to the Zelen method [51]. During the inclusion visit, patients will be informed that the purpose of the research is to study the effectiveness of the care provided during spa therapy. The content of spa therapy will not be explained in detail right away and randomization will occur before consent is obtained. In the event of refusal after allocation of the intervention group (e.g., the patient not wishing to undergo care for 6 weeks), the patient will be assigned to the other group. An information letter and a consent specific to each group will be proposed.

Randomization will be performed centrally and independently by use of a computer program at the Centre for Clinical Epidemiology (CIC EC). The randomization group will be available during the selection visit.

(b) Blinding methods.

The assessor will be blinded to the treatment group at 3 and 6 months.

**4.4. EXPERIMENTAL DRUGS / MEDICAL TREATMENTS UNDER STUDY**

NA

**4.5. EXPECTED DURATION OF PARTICIPATION OF PERSONS AND DESCRIPTION OF THE CHRONOLOGY AND DURATION OF ALL TRIAL PERIODS, INCLUDING FOLLOW-UP, IF NECESSARY.**

Inclusion visit

The investigating physician verifies that the patient meets the inclusion criteria defined in advance, during a consultation carried out in the rheumatology department of Nancy University Hospital, Vandoeuvre les Nancy.

This physician will ensure that the patient has a knee X-ray performed according to current recommendations to verify the eligibility criteria. If necessary, a digitized radiograph will be taken in the rheumatology department.

After randomization, the investigating physician gives the eligible patient the information form that corresponds to the type of cure selected and obtains his/her consent to enter the study.

A plan is given to the patient.

Follow-up visits

Subsequent visits will all take place at the Nancy Thermal site in the spa center. They will involve the thermal physician and/or the assessor who will collect the data necessary for the study (V0, V1, V2, V3 and V4) (see monitoring table). The thermal physician also ensures the medical follow-up of the treatment and collects adverse reactions.

Occupancy, group planning:

This study will be conducted from mid-March to mid-December 2012. The flow of patients has been planned for both types of treatment: "classic spa therapy", “active spa therapy". A subgroup will consist of a maximum of 8 patients. Over 6 weeks and for each type of spa therapy, 4 subgroups can be included, i.e., 32 patients randomized in each group (64 patients). The study will focus on 5 cycles of 6 weeks for a total time of 30 weeks.

In the morning, the premises, care and carers will be dedicated to the "classic spa therapy" group, i.e., a daily occupation over 3 consecutive weeks for 2 subgroups of patients or over 6 weeks for 4 subgroups of patients.

In the afternoon, the premises, care and carers will be dedicated to the group “active spa therapy" for 3 weeks of usual spa therapy, then 3 weeks of rehabilitation/education by alternating sub-groups on alternate days, i.e., a daily occupation over 6 weeks for 4 subgroups.

This organization will allow for the same premises for both groups ("classic spa therapy" and “active spa therapy") while avoiding that they are in close contact, comparing their care while avoiding shifting the groups over time.

Coordination

Coordination, logistics management, quality control, data management, and statistical data processing will be carried out by the CIC EC in Nancy.

Expected inclusion period: from January 2012 to the end of October 2012, i.e., 10 months

Duration of participation (including follow-up period): for one patient, 6 months from the beginning of the spa therapy

Total duration (including data analysis time): December 2011 to mid-October 2013 or 2 years

|  | **Selection inclusion** | **V0** | **V1**  **3** weeks | **V2**  **6** weeks | **V3**  **3 months** | **V4**  **6 months** |
| --- | --- | --- | --- | --- | --- | --- |
| Inclusion  Zelen randomization  Consent | X  X  X |  |  |  |  |  |
|  |  |  |  |  |  |  |
|  |  |  |  |  |  |  |
| Sociodemographic data | X |  |  |  |  |  |
| History of OA | X |  |  |  |  |  |
| Clinical examination | X |  |  |  |  |  |
| X-ray | X |  |  |  |  |  |
| Ultrasonography | X |  |  |  |  |  |
| Treatment | X | X | X | X | X | X |
| VAS pain | X | X | X | X | X | X |
| Clinical follow-up |  | X | X | X | X | X |
| WOMAC |  | X | X | X | X | X |
| Quality of life |  | X | X | X | X | X |
| Posturography |  | X | X | X | X | X |
| 6-minute walking test |  | X | X | X | X | X |
| Care consumption |  | X | X | X | X | X |
| Adverse reactions |  |  | X | X | X | X |
| Satisfaction, heiQ |  |  | X | X | X | X |

**4.6. PROCEDURES FOR ACCOUNTING FOR EXPERIMENTAL MEDICINAL PRODUCTS**

NA

**4.7. PROVISIONS IMPLEMENTED TO MAINTAIN BLINDING AND** PROCEDURES **TO REMOVE THE BLINDING, IF NECESSARY.**

NA

**4.8. IDENTIFICATION OF ALL DATA TO BE COLLECTED DIRECTLY IN THE OBSERVATION WORKBOOKS, WHICH WILL BE CONSIDERED AS SOURCE DATA.**

**Assessment tools**

Data collection will be carried out by the investigating physician at the time of inclusion then by the thermal physician and the assessor at other times.

Sociodemographic and clinical examination data:

The following demographic and clinical characteristics will be collected: age, sex, socioeconomic data, risk factors associated with knee OA, comorbidity index, drug, clinical examination (weight, height, static lower -limb disorders, uneven length of the lower limbs, joint effusion, meniscal test, mobility of the knee joint, examination of the hip and spine, 6-minute walking test). The 6-minute walking test is easy to implement and allows for assessing the direct impact of OA on walking [56], judging the effectiveness of a valgisation orthosis in knee OA [57] and for predicting the effectiveness of total hip arthroplasty [58].

Patient-reported outcome measures

WOMAC index

It will be used as the basis for calculating the main outcome criterion. It was developed by Bellamy in 1988 [60] and is now more widely used than the Lequesne index and has a good sensitivity to change [61].

VAS-Pain

Pain measurement will be measured on a VAS according to Huskinsson [62]. We will use one of the recommended methodologies by the French High Authority for Health.

Quality of life (QoL)

The SF-36 is a generic quality of life instrument that has been widely used since 1992 [63]. It was recently used to evaluate the effectiveness of spa treatment during knee OA [64]. OAKHQOL is a QoL instrument specific for knee and/or hip OA [3]. This tool is used to evaluate the effectiveness of treatment during knee OA (viscosupplementation, total joint replacement surgery)[65, 66].

X-rays

Each patient included should have knee X-rays < 6 months old with a femoro-tibial incidence in Schuss position and a femoral-patellar incidence of 60°. A radiographic evaluation according to the Kellgren and Lawrence classification will be performed by the investigating physician during the inclusion visit [5].

Ultrasonography

This examination is carried out by the investigating physician. It allows for classifying knee OA in congestive or non-congestive forms at inclusion. This non-radiant and non-traumatic exam will help to appreciate the importance of joint effusion and synovitis and to objectify the presence of a popliteal cyst by using a score validated for the first time in this type of population [6]. Joint effusion and synovitis will be evaluated on 3 regions of interest: median, medial and lateral quadricipital ramps. The vascular activity of synovitis will be explored in power Doppler mode.

The presence of a popliteal cyst will be sought in the ventral decubitus.

The effusion varies from 0 to 9 and will be considered positive at ≥ 1. The synovitis score ranges from 0 to 15 and will be considered positive at ≥ 1.

The presence of a popliteal cyst will be rated binary: present or absent [6].

Posturography

The posturography examination will be the responsibility of Professor P. Perrin and his team. The examination will be conducted by using a movable platform in a room with sound insulation to avoid interference. The posturography examination is performed without and then with the use of glasses of virtual reality that will distort vision (eyes open/closed/vision distorted) in static and then dynamic situations (foam or swashplate).

At least 6 data points will be acquired. The examination will assess the components proprioceptive, visual and vestibular that come into play in the individual's postural balance [4]. Proprioceptive control appears to fail during knee OA [11], but this study will make it possible to measure the impact of the classical and active spa therapy by measuring the change in length travelled and/or the area covered by the foot pressure centre after the suppression or disruption of sensory inputs and/or somesthesics during treatment and follow-up[ 4]. A recent review showed that active rehabilitation improves control proprioceptive [59].

**The main and secondary evaluation criteria**

Main criterion

# The MCII in OA was determined by Tubach et al. [2]. The MCII is a threshold for improvement which is particularly suitable for therapeutic trials and takes into account that the MCID may be different for an improvement or a worsening. A patient is considered improved if the WOMAC index function subscale score is improved by at least 9.1 from the initial value and/or if the current pain measured on a VAS is improved by at least 19.9 mm. These values correspond to the smallest clinically relevant change for these two outcome criteria [2]. Improvements below this value will be considered treatment failures. Patients with improvement but who still need surgery during the period of the study will also be considered failures. The effect of two types of spa therapy ("standard spa therapy" and "active spa therapy") for symptomatic knee OA will be assessed by proportion of patients who have achieved a composite criterion combining the MCII for pain and/or functional abilities and the absence of knee surgery of the target knee at 6 months.

Secondary criteria

- Percentage of patients reaching MCII (pain, function and composite score) at the end of the treatment and at 3 months
- Percentage of patients reaching the PASS (pain and function) at 3 and 6 months [14]. The PASS is the value beyond which the patient considers himself/herself to be well. It is defined as for the MCII by the 75th percentile of the score among the patients whose assessment of the acceptable symptom state was “good” on a Likert scale with 5 answer modalities. The PASS has been determined for WOMAC function at 31 mm and for VAS for pain at 32 mm [14].
- Evolution of quality of life scores (OAKHQOL, SF-36) at 3 and 6 months
- Evolution of VAS-fatigue, VAS-pain at 3 and 6 months
- Evolution of WOMAC scores (function) at 3 and 6 months
- Patient satisfaction (overall VAS or heiQ module 9) at 3 and 6 months
- Evolution of drug and care consumption, use of surgery
- 6-minute walking test [43]
- Evolution of posturography parameters: modification of the length travelled and/or the area covered by the pressure centre of the feet after the removal or disruption of sensory inputs, somesthesics of balance in distorted visual situations, and static and dynamic positions [4]
- Adverse reactions

**4.9. COLLECTION OF BIOLOGICAL SAMPLES**

NA

**5. INCLUSION AND EXCLUSION OF PEOPLE FROM RESEARCH:**

5.1. INCLUSION CRITERIA

The patients with knee OA:

- Meeting the criteria for knee OA of the ACR (*)
- Uni- or bilateral OA
- Symptomatic: VAS pain > 3 on a scale of 0 to 10
- Radiological femoro-tibial knee OA³ stage 2 of the Kellgren and Lawrence classification
- With consent
- Preliminary medical examination

* The diagnostic criteria used are those of the ACR [Altman 1986]:

- Knee pain and at least 1 of the following three criteria:
  - Age > 50 years old
  - Morning stiffness < 30 min
  - Crepitus on active motion
- The presence of osteophytes on knee X-rays

(sensitivity 94%, specificity 87%)

5.2. NON-INCLUSION CRITERIA

- Isolated patellofemoral OA
- Indication for total joint replacement surgery within 1 year
- Contraindication to spa therapy (immune deficiency, moderate to severe heart failure, cancer, infection, skin wound etc.) or predictable intolerance to spa therapy (intolerance to heat, baths, hot tubs, swimming pool etc.)
- Other disease of the musculoskeletal system (inflammatory arthritis, disabling lumbar radiculopathy, motor deficit of the lower limbs)
- Severe comorbidities
- Severe depressive syndrome, psychosis
- Previous knee treatments:
  - Massages, physiotherapy, acupuncture of M 1 month
  - NSAID change within the last 5 days
  - Change of analgesics within the last 24 hr
  - Change of symptomatic slow-acting drugs in OA of < 3 months
  - Intra-articular injection of the target joint of < 1 month for corticoids and < 3 months for viscosupplementation
  - Physiotherapy or rehabilitation of < 1 month.
  - Spa therapy during the previous year

**5.3. PROCEDURE FOR EARLY TERMINATION OF THE RESEARCH OR EXCLUSION (DISCONTINUATION OF TREATMENT + DISCONTINUATION OF FOLLOW-UP)**

**5.3.1. CRITERIA AND PROCEDURES FOR STOPPING PROCESSING / AND EXCLUSION**

- FINAL STOP: In case of a serious adverse reaction

- No prohibition to participate in another protocol

**5.3.2. DATA COLLECTION AND MONITORING PROCEDURES**

The follow-up visits will all take place at the Nancy Thermal site in the spa center. They will be carried out by the thermal physician and/or the assessor who will collect the data necessary for the study (V0, V1, V2, V3 and V4) (see monitoring table). The thermal physician also ensures the follow-up medical treatment and collects adverse reactions.

**5.3.3. REPLACEMENT OF PERSONS**

NA

**5.3.4 PROCEDURES FOR MONITORING PEOPLE**

NA

**5.4. RECRUITMENT PROCEDURES**

Information on the Nancy Thermal study will be provided to general practitioners and rheumatologists (mail and/or information meeting) in the months preceding the start of the study. Patient information will be provided through the press (regional press release and local radio stations), as well as by a poster campaign in the offices of physiotherapists, pharmacies, biological laboratories, and in the waiting rooms of general practitioners and rheumatologists. A call centre will be set up to plan appointments with investigating physicians.

Patients, informed by press and poster and wishing to participate, will make an appointment by telephone with the call centre (toll-free number put into service in December 2011). This first date will correspond to their selection visit. General practitioners and rheumatologists informed by letter/information meetings may refer their patient via the call centre.

**6. INTERVENTION**

6.1. Care protocol

"Classical spa therapy".

The care protocol strictly follows the one recently validated in the "Thermarthrosis" study[1], i.e., 4 thermal treatments per day, 6 days a week for 3 weeks (18 sessions in total).

The 4 thermal treatments are (1) water jet at 37°C for 15 min in individual sessions; (2) massages under jet at 38°C by physiotherapist for 10 min, (3) Illutations (sludge applications) at 45°C for 15 min, (4) mobilization in the pool at 32°C in collective sessions (groups of 6) for 25 min.

"Active spa therapy"

This cure combines successively 3 weeks of thermal treatments according to the Thermarthrosis scheme (4 cares/sessions) but at the rate of 3 sessions per week (9 sessions) then 3 weeks of rehabilitation/active physiotherapy at 3 sessions per week (9 sessions) for a total of 6 weeks and a total of 18 sessions.

This approach aims to meet the following objectives: to prolong the indolence acquired in phase 1, to improve joint mobility and muscle function, improve proprioception and develop appropriate attitudes and behaviour. Indeed, after a rest phase, it seems logical to recondition the patient for effort by correcting proprioceptive disorders and muscle deficiencies to prolong the long-term effectiveness of the spa treatment. However, no study has so far assessed the value of combining a phase of sedation provided by thermal treatments with an active approach of muscle strengthening and proprioceptive work in knee OA.

The first phase of active spa therapy includes the 4 treatments of classic spa therapy at the rate of 3 sessions per week for 3 weeks, i.e,. 9 sessions: waterjet at 37°C for 15 min in these individual sessions, massages under jet at 38°C by physiotherapist for 10 min, illutations (sludge applications) at 45°C for 15 min and mobilization in the pool at 32°C in group sessions (groups of 6) for 25 min [1].

The second phase of care was defined in collaboration with Professor J Paysant, and the persons in charge of the School of Physiotherapy, Mrs R. Ceconello and P Boisseau. Practical care will be provided by students in massage and physiotherapy under the supervision of a qualified professional physiotherapist. It will take place over 3 weeks at the rate of 3 sessions per week, or 9 sessions.

Rehabilitation care will include the following:

1) preparatory work by the lower limbs based on local hot physiotherapy ("hot pac"), the participant being in a strict supine position with cushions under the head and under the popliteal cavity of the non-massaged knee, and manual massages with effleurage of the lower limb, sliding pressure or static pressure stepped from the root of the lower limb to the foot and back for deep and superficial venous system, maneuvers relaxing thigh, calf and foot muscles followed by circulating sliding pressures. This treatment can be completed with palpating-rolling in the peri-articular region of the knee and a global effleurage for a total duration of 20 min;

2) a personalized work consisting of musculo-tendinous stretching adapted to the initial assessment, carried out in stages or continuously, manually or by posture, followed by muscle strengthening, preferably static or in a chain, always adapted to the initial assessment and carried out against manual resistance, of the diagonal spiral Kabat type, or instrumental resistance, of the elastic, analytical or global resistance type;

3) global work, proprioceptive and functional, consisting of tests and eye balance exercises with eyes open and closed, on a Satel or Biorescue rehabilitation platform and on carpets and locomotion work on standardised walking paths;

4) a group educational session according to a predefined program with information about the disease, drug and non-drug OA management, risk factors (activities and weight), footwear and technical aids, lifestyle advice and exercises to be done at home and the hand-delivery of a follow-up booklet.

In the middle of the course, these sessions will be based on question-and-answer questionnaires, learning self-exercises and learning attitudes and gestures to be practiced in high-risk situations. At the end of the cure, these sessions will be conducted to self-correct exercises and gestures as well as the implementation of a physical activity program to be continued.

Physiotherapy care will be individualized and adapted (type and intensity of the exercises) to the initial physiotherapy assessment according to the recommendations of SOFCOT (Society for Orthopaedic Surgery) and SOFMER (Society of Medicine and Rehabilitation) from 2007.

Course of the spa therapy

The spa therapy will be carried out in the round pool, Jacques Baudot esplanade, 43 rue du Sergent Blandan, 54000 Nancy. A space devoted to this study has been fully brought up to standard by the company “Thermes experts/ EUROSPA”. This space includes a reception area, a waiting room, a personal room, a medical office, a cloakroom for individuals taking the waters, a relaxation room, a physiotherapy and rehabilitation room, 2 shower rooms, 2 bathrooms, 2 affusion rooms, a water jet cabin, a hygiene plant, a sludge manufacturing room, a laboratory room, toilets and a laundry room.

A request for provisional approval of the use of water and premises for thermal care will be deposited with the Academy of Medicine after authorization from the AFSSA (stable bacteriological and chemical samples for a period of 12 consecutive months: in progress since December 2010) and the Prefect of the department of Meurthe et Moselle.

The chemical qualities of sodium chloride water are identical to those of the thermal spa of Amnéville les Thermes.

Spa therapy will be provided by physiotherapist professionals of spa and physiotherapy/rehabilitation care. They will be responsible for carrying out care, supervising care and monitoring tolerance and compliance with treatment. A training session will be provided prior to the start of the study to standardize practices.

The thermal physician will be in charge of examining the treatment to patients before the spa therapy, during the course of spa therapy and at the end of the spa therapy to ensure that it runs smoothly. The patients will continue their usual treatment.

**6.4. METHOD OF MONITORING COMPLIANCE**

The number of treatments actually performed by patients will also be recorded by the thermal doctor

**7. EFFECTIVENESS EVALUATION**

**7.1. DESCRIPTION OF THE PARAMETERS FOR EVALUATING EFFECTIVENESS**

Main Criterion

# The MCII in OA was determined by Tubach et al. [2]. The MCII is a threshold for improvement that is particularly suitable for therapeutic trials and takes into account that the MCID may be different for an improvement or a worsening. A patient is considered improved if the WOMAC index function subscale score is improved by at least 9.1 from the initial value and/or if the current pain measured on a VAS is improved by at least 19.9 mm. These values correspond to the smallest clinically relevant change for these two outcome criteria [2]. Improvements below this value will be considered treatment failures. Patients with improvement but would still need to have surgery during the study period will also be considered failures. The effect of the two types of spa therapy ("standard spa therapy" and "active spa therapy") for symptomatic knee OA will be assessed by proportion of patients who achieve a composite criterion combining the MCII for pain and/or functional abilities and the absence of knee surgery of the target knee at 6 months.

Secondary criteria

- Percentage of patients reaching the MCII (pain, function and composite score) at the end of the treatment and at 3 months
- Percentage of patients reaching the PASS (pain and function) at 3 and 6 months [14]. The PASS is the value beyond which the patient considers himself/herself to be well. It is defined as for the MCII by the 75th percentile of the score among patients whose assessment of the acceptable symptom state was “good” on a Likert scale with 5 answer modalities. The PASS for WOMAC function is 31 mm and for VAS for pain is 32 mm [14].
- Evolution of quality of life scores (Osteoarthritis Knee and hip Quality of life [OAKHQOL], SF-36) at 3 and 6 months
- Evolution of VAS-fatigue, VAS-pain at 3 and 6 months
- Evolution of WOMAC scores (function) at 3 and 6 months
- Patient satisfaction (overall VAS or heiQ module 9) at 3 and 6 months
- Evolution of drug and care consumption, use of surgery
- 6-minute walking test [43]
- Evolution of posturography parameters: modification of the length travelled and/or the area covered by the pressure centre of the feet after removal or disruption of sensory inputs, somesthesics of balance in distorted visual situations and static and dynamic positions [4]
- Adverse reactions

**7.2. METHODS AND SCHEDULE FOR MEASURING, COLLECTING AND ANALYSING THE PARAMETERS FOR EVALUATING EFFECTIVENESS.**

Clinical and perceived health data will be collected before the cure (V0), at 3 weeks (V1: end of the classic spa therapy or change of type of care for active spa therapy), at 6 weeks (V2: end of active spa therapy), at 3 months (V3: M3) and 6 months (V4: M6) and will include the following examinations: clinical exam (knee effusion, joint amplitudes), VAS-pain, VAS-fatigue, WOMAC, OAKHQOL, SF-36, satisfaction, drug consumption, total replacement surgery, side effects, posturography examination, 6-minute walking test. The number of spa care actually performed by the patients will also be listed (see follow-up table).

**8. SAFETY ASSESSMENT:**

**8.1. DESCRIPTION OF THE SECURITY ASSESSMENT PARAMETERS**

Adverse reactions that can be experienced with the spa therapy are rare, most often minor and transient. They are traditionally observed during the period of the spa therapy and are then part of what is usually called the "thermal crisis": tiredness, waking up of some of the usual pain, unusual sweating and insomnia. These effects rarely require the cessation of spa therapy. More serious health problems are possible but remain exceptional: heart problems, bacterial infections that may be present in warm waters (including Legionella infection).

Adverse reactions are noted at each follow-up visit.

No systematic biological or imaging monitoring of secondary effects is necessary.

**8.2. METHODS AND SCHEDULE FOR MEASURING, COLLECTING AND REPORTING THE SECURITY ASSESSMENT PARAMETERS**

The health care professionals performing spa therapy and the thermal physician are empowered to supervise patients as part of the management of the spa therapy.

Adverse reactions are noted at each follow-up visit.

Emergency contact information will be provided to patients:

Pr I Chary-Valckenaere,

Rheumatology Department

Nancy University Hospital, Brabois Hospitals;

Tel. 03 83 15 32 93,

Fax 03 83 83 15 31 90,

[rhumato.secretariat3@chu-nancy.fr](mailto:rhumato.secretariat3@chu-nancy.fr)

**8.3 PROCEDURES IN PLACE FOR THE REGISTRATION AND NOTIFICATION OF ADVERSE EVENTS**

**8-3-1 Definitions**

An adverse event is a harmful event that occurs in a person who is engaged in biomedical research, whether this person is related or not to the research or product to which this research relates.

An adverse reaction is a harmful and unintended reaction to an experimental drug, occurring at the normal dosage used in humans.

A serious adverse event (SAE) or serious adverse reaction is an event or effect that may have contributed to the patient's death, life-threatening event, hospitalization or prolongation of hospitalization, incapacity or significant disability, or lasting disability, or result in a congenital anomaly or malformation.

The list of expected adverse events related to the study is based on of the registration document.

An SAE is unexpected when it is not on this list.

**8-3-2 Reference document to define the expected character of an SAE**

NA

**8-3-3-3 List of expected adverse reactions**

Adverse reactions that can be experienced with the spa therapy are rare, most often minor and transient. They are traditionally observed during the period of the spa therapy and are then part of what is usually called the "thermal crisis": tiredness, waking up of some of the usual pain, unusual sweating and insomnia. These effects rarely require the cessation of spa therapy. More serious health problems are possible but remain exceptional: heart problems, bacterial infections that may be present in warm waters (including Legionella infection).

**8-3-4 Transmission of SAE**

As soon as an investigator becomes aware of a SAE or a new event, he/she shall declare this without delay to the promoter by faxing the SAE declaration form at 03 83 32 32 33 44.

- If it is an unexpected serious adverse reaction or a event, the sponsor will contact the investigator to write a report, which will be forwarded to the AFSSaPS, CPP and EUDRAVIGILANCE within 7 days in the event of death or vital prognosis, if not within 15 days.

When the event is not resolved on the date the fax is sent, the investigator is required to send a supplementary report to document the evolution or update missing data.

- An expected SAE will be collected by the sponsor for the preparation of annual safety reports.

**8-3-5 Transmission of non-serious adverse reactions**

These will be briefly described by the investigator on the summary sheet dedicated to this end.

**8.4. MODALITIES AND DURATION OF FOLLOW-UP OF PERSONS FOLLOWING THE OCCURRENCE OF UNDESIRABLE EVENTS**

When a serious adverse event persists, including after the end of the study, the investigator will follow the patient until the event is considered resolved and will forward the follow-up data to the appropriate body.

**8.5. SPECIFIC RESEARCH COMMITTEES**

**8.5.1 STEERING COMMITTEE**

Mr. Ceconnello, Pr I. Chary-Valckenaere, Pr F Guillemin, Pr D. Loeuille, Pr J Paysant, Dr. AC Rat, Dr. V. Royaux, Dr. S Ruscade-Cholay.

It will define the general organization and conduct of the research and will coordinate the information. It will initially determine the methodology and decide during the research process procedures to be followed in unforeseen cases and will monitor the progress of the research in particular in terms of tolerance and undesirable events.

**8.5.2 INDEPENDENT SUPERVISORY COMMITTEE – DATA SAFETY MONITORING BOARD**

Pr P Gillet, Pr F Tubach (Methodologist), Pr X Chevalier, Pr C. Tavernier

An independent Supervisory Committee has an advisory and decision-making function when called upon by the proponent on medical issues such as tolerance and adverse events. It consists of people from outside the research, necessarily one of whom is a clinician specializing in the pathology studied and a pharmacologist/pharmacovigilant and according to the protocol a methodologist/biostatistician.

**8.5.3 INDEPENDENT CRITICAL EVENT EVALUATION COMMITTEE**

NA

**8.6 SAFETY REPORTS**

- Annual Safety Reports: the sponsor prepares the annual safety reports and forwards them to the AFSSaPS, CPP. The coordinating investigator will forward to the sponsor all the data necessary for the preparation of this report.

- Final report: written by the sponsor and the coordinating investigator within 1 year after the end of the study. All investigators are informed of the results of the study. A summary is sent to the AFSSaPS by the promoter.

**9. STATISTICS**

**Number of subjects required**

- Main objective: with a 50% percentage of responders to standard spa therapy at 6 months [1], a power of 80%, and an alpha risk of 5%, 135 patients per group are required to significantly demonstrate the non-inferiority of the active spa therapy with a lower confidence limit of the difference in proportions of 15%. Considering that the number of patients lost to follow up may be limited to 5%, a total of 284 patients should be included in the trial.
- For the secondary objective of comparing 3-month responders, the percentage of responders being higher than at 6 months, the number of subjects needed is lower: with a 60% percentage of responders to standard spa therapy at 3 months, a power of 80%, an alpha risk of 5%, 77 patients per group are required to significantly demonstrate the non-inferiority of the active spa therapy with a lower confidence limit of the difference in proportions of 19%. For demonstrating an effect of spa therapy sufficient to obtain thermal approval, the evidence of an effect size of 0.3 in an analysis of the before-and-after effect in the same group [6] with 80% power and 5% alpha risk with a test, 90 patients are needed in each group.

**Descriptive statistics**

Qualitative criteria will be described by their number and percentage and quantitative criteria by average, standard deviation or median and interquartile range (25th percentile- 75th percentile).

Results after treatment will be described by the number and percentage of responders according to the composite criterium, MCII pain, MCII function, PASS criteria and by the evolution of scores (means, standard deviation of differences) of self-questionnaires measuring health status.

To compare the evolution before and after treatment, matched t-tests will be carried out for quantitative assessment criteria and by testing McNemar for binary outcome criteria.

**Comparison of groups**

The trial being a non-inferiority trial, the main analysis will be per protocol to be conservative by increasing the contrast between the groups.

Main objective

The active spa therapy will not be considered inferior to the standard spa therapy if the lower bound of the confidence interval at 90% of the difference between the 2 groups (active spa therapy minus standard spa therapy) for the main outcome criterion (percentage of respondents according to the composite criterion) is not less than 15%[1]. After verification of the randomization that could be unbalanced owing to the Zelen randomization mode, the final analysis will be adjusted if necessary on the variables that differ between the 2 groups.

Secondary objectives

- The same analyses will be repeated for the MCII composite criterion, pain and function at 3 months and for PASS criteria.

- To determine the predictive factors of spa therapy response, a bivariate analysis will be performed using chi-square tests (or Fisher exact tests depending on the number) for discrete variables, and Student t-tests, ANOVA or Wilcoxon tests for quantitative variables. The parameters significant on bivariate analyses (at the conservative threshold of p=0.1) will then be introduced into a stepwise multiple logistic regression.

A value of p<0.05 will be considered statistically significant.

The statistical analysis will be carried out by the Centre for Clinical Epidemiology by using SAS v9.2 (SAS Inc., Cary, NC).

**10. RIGHT OF ACCESS TO SOURCE DATA AND DOCUMENTS**

All data and information concerning the patient will remain strictly confidential. Persons with direct access in accordance with the laws and regulations in force, in particular Articles L.1121-3 and R.5121-13 of the Public Health Code (e.g., the investigators, quality control persons, monitors, clinical research assistants, auditors and others called upon to collaborate in the trial) will take all necessary precautions to ensure the confidentiality of information relating to experimental treatment, testing, and those who lend themselves to it, and in particular with regard to their identity and the results obtained. The data collected by these persons during quality controls or audits are then made anonymous.

**11. QUALITY CONTROL AND ASSURANCE**

**11.1 Data quality control**

A Clinical Research Assistant mandated by the Nancy DRI will regularly perform monitoring visits in the investigation centre. On this occasion, this person should have access to the observation books, the medical records and all study documents. This person will check adherence to the protocol as well as the compliance with document sources and data consistency.

**11.2 Monitoring of the centres**

Monitoring in the investigation centre will be regular according to the rate of inclusions and a monitoring plan established by the proponent.

**12. ETHICAL AND REGULATORY ASPECTS**

**12.1 INFORMATION DOCUMENT AND INFORMED CONSENT**

Patients will be able to participate in this study only if they have given their written consent.

Information

Beforehand, they will have received oral and written information from the physician investigator on the purpose of this study, the duration of their participation, the procedures, the benefits, the foreseeable risks, the confidentiality of the data, insurance coverage, coverage of costs related to the trial. All this information will be summarized in an information document given to every patient.

Informed consent

The consent form will be signed in duplicate by the participant and the physician investigator. This document will be given to the patient. The investigator should keep the second copy for a minimum of 15 years.

**12.2 DATA CONFIDENTIALITY**

All data collected will be treated anonymously and covered. by medical confidentiality.

The investigator will ensure that patient confidentiality is maintained. In observation notebooks, patients will be identified by the first initial of the name, the first initial of the first name and the inclusion number.

**12.3 LAWS AND BPC**

The investigators undertake to ensure that this study is carried out in accordance with law no. 2004-806 of August 9, 2004, as well as in accordance with Good Clinical Practices (I.C.H. version 4 of May 1, 1996 and decision of November 24, 2006) and the Declaration of Helsinki (Ethical Principles for Medical Research on Subjects Seoul 2008). To do this, a study contract will be dated and signed between the principal investigators from each participating centre and the sponsor.

**12.4 SUBMISSION OF THE PROTOCOL TO THE CPP AND THE COMPETENT AUTHORITY**

The study sponsor will submit this protocol to CPP EST III, in accordance with the legislation in force of the Public Health Code. The sponsor will request also the authorization of the study to the AFSSAPS before the start of the project.

**12.5 PROTOCOL MODIFICATION**

There will be no change to this protocol without the agreement of the sponsor. The proponent will qualify this change as substantial or not. Any substantial modification must be submitted to the CPP and/or AFSSAPS before its implementation.

**12.6 COMPUTERIZATION OF DATA**

In accordance with the Data Protection Act, the files and freedoms in force, the patient has a right of access, communication, opposition and rectification of personal information, which is provided upon written request to the coordinating investigator.

This research is part of the "Reference Methodology" (MR-001) pursuant to the provisions of article 54 paragraph 5 of the law of January 6, 1978 modified relating to data processing, files and freedoms. This change has been approved by decision of January 5, 2006. NANCY University Hospital signed a commitment to comply with this "Reference Methodology".

**12.7 CURRICULUM VITAE AND SIGNATURE OF THE PROTOCOL**

Before starting the study, the coordinating investigator will provide representatives of the sponsor a copy of his personal CV dated and signed as well as those of the associate investigators.

**12.8 PROMOTION**

The promotion of this trial is ensured by the NANCY University Hospital.

**12.9 STUDY REPORT AND PUBLICATIONS**

The final report of the research will be written by the coordinator and the person responsible for data analysis. This report will be submitted to each of the investigators for their opinion. Once a consensus is reached, the final version must be endorsed by the signature of each of the investigators and sent to the sponsor as soon as possible after the effective end of the search.

Nancy University Hospital is the owner of the data and use or transmission to a third party may not be carried out without its prior consent. NANCY University Hospital must be mentioned as the biomedical research sponsor.

**12.10 FILING AND ARCHIVING**

Research documents falling within the scope of the Research Act biomedical records must be archived by all parties for 15 years after the end of the research. This indexed archiving must include all documents related to the research.

**13. FINANCING**

Budget

This part takes into account the additional costs incurred by the CHU in the service of Rheumatology and CIC-EC INSERM, for the Training Institute in Massokinesitherapy, and for the Department of Physical Medicine and Rehabilitation

Rheumatology Department

Missions: development of the research program, communication, recruitment, data collection (inclusion visit), interpretation of data.

Project preparation: organization of scientific meetings, drafting of the protocol and CRF, coordination with partners: CUGN, CIC EC INSERM, Physical Medicine and Rehabilitation, sponsor; study planning;

Data collection: patient inclusion data

Analysis and interpretation: interpretation, writing report for thermal approval and scientific article

Estimated budget Rheumatology (euros)

|  | Year **2011** | Year **2012** | Year **2013** | **Total** |
| --- | --- | --- | --- | --- |
| Preparation of the draft of the research - Writing of the protocol - Writing a report | 2500 |  | 2500 |  |
| Investigating physician: 98 euros per patient (400 patients examined) |  | 39 200 |  |  |
| Digitized radiographs  19.95 euros per patient (n=400) |  | 7 980 |  |  |
| Computer equipment: Laptop computer, external hard disk backup and camera | 2500 |  |  |  |
| Total | 5000 | 39200 | 2500 | 46700 |

Thermal physician(s):

Missions:

Follow-up of patients during spa therapy,

Adverse reaction reporting

Data collection and evaluation at V0 and V1

Estimated budget: estimated budget 1.5 full-time equivalents March 2012 to December

2012: 100,000 euros

CIC-EC INSERM

Missions: Management of the research project - collection, analysis and interpretation of the data

Project preparation: regulatory dossiers: CPP, AFSSAPS;

Collaboration with the promoter; coordination with partners: CUGN, Rheumatology, rehabilitation; definition and traceability of the patient circuit; coordination meetings; preparation of the CRF; opening of a toll-free telephone line; communication documents; preparation of the centralized randomization; construction of a study management database; database construction; database and document tests of observation; printing of the CRF.

Data collection: Collection sites: inclusion, visits; quality control, monitoring of the study (promoter)

Analysis and interpretation: data management; statistical analysis, writing an analysis report, interpretation.

Resource requirements

Staff members

Epidemiologist: 6 months preparation (0.2 full-time equivalent) - project follow-up 12 months (0.1) – 3 month analysis interpretation (0.2)

Project manager: 6 months of preparation (0.5) - 9 months project follow-up (0.2) – 6 month study report, communication to patients (0.5)

Secretary: 21 months (0.2 x 6 months + 0.2 x 3 months)

Clinical research asssociate: patient management and data collection: 3 months preparation specification documents (0.3) - 3 months preparation recruitment (0.3) - 9 months monitoring and collection of data (0.5)

Data entry technician: 9 months (0.1)

Database and statistics engineer: 3 months construction (0.2) - 9 months data management (0.1) - 3 months statistical analysis and statistical report (0.3)

Functioning

Printing of CRF

Laptop computer

Small supplies

Travel in urban areas

Estimated budget

| Ressources | Year 2011 | Year 2012 | Year 2013 | Total |
| --- | --- | --- | --- | --- |
| *Sponsoring* |  |  |  |  |
| Insurance | 800 |  |  | 800 |
|  |  |  |  |  |
| Ethics, regulatory submissions | 800 |  |  | 800 |
| (subtotal 1) | *1600* |  |  | *1600* |
|  |  |  |  |  |
| Staff members |  |  |  |  |
| Epidémiologist | 5000 | 5000 | 2500 | 12500 |
| Project manager | 5000 | 11000 | 10000 | 26000 |
| Clinical research associate 0.75 full-time equivalent | 3500 | 31500 |  | 35000 |
| Nurse of research clinical (follow-up 6 weeks, 3 and 6 months) of 300 patients 1 full-time equivalent |  | 50000 |  | 50000 |
| Input technician | 0 | 3000 | 0 | 3000 |
| Secretary | 1800 | 1800 | 1800 | 5400 |
| Engineer based in data - biostatistician | 2250 | 3400 | 3300 | 8950 |
| (subtotal 2) | *17550* | *105700* | *17600* | *140850* |
|  |  |  |  |  |
| *Functioning* |  |  |  |  |
| Printing | 5000 |  |  | 5000 |
| Laptop computer | 1200 |  |  | 1200 |
| Travel, phone | 500 | 2000 | 500 | 3000 |
| Small equipment | 500 | 200 | 500 | 1200 |
| (subtotal 3) | *7200* | *2200* | *1000* | *10400* |
| (subtotal 1+2+3) | *26350* | *107900* | *18600* | *152850* |
|  |  |  |  |  |
| Financial expenses |  |  |  |  |
| Structure costs  CIC-EC (10%) | 2635 | 10790 | 1860 | 15285 |
| Promoter Fees (management, monitoring) (10%) | 2898,5 | 11869 | 2046 | 16813,5 |
| (subtotal 4) | *5533,5* | *22659* | *3906* | *32098,5* |
| Total (1+2+3+4) | 31883,5 | 130559 | 22506 | 184948,5 |

Patients will be compensated for their transport according to a fixed price.

Physiotherapy school and physical medicine department and Rehabilitation

Physiotherapist trainees (completion of phase 2 of the active spa therapy) do not receive a remuneration over the academic period; they will in fact be partially assigned to the study from their internship field. However, during school holidays, physiotherapist trainees will receive remuneration according to the rates in force, i.e,. the gross monthly minimum wage of 1365€; for 8 part-time physiotherapist students for 2 months, the cost total is 10 920€.

They will be supervised by a physiotherapist from the Training Institute in Masso-Kinesitherapy (hourly cost = 44.37€ in charge of social taxes).

Over 6 weeks, 4 groups of 8 patients will be treated at a rate of 9 afternoons of 5 hours. Either 90 hours per cycle or a total cost for the 5 cycles of 450 hours x 44.37 = 20,000 euros

Reception and maintenance staff

These agents are part of Nancy Thermal's staff

**REFERENCES**

1 Forestier R, Desfour H, Tessier JM, Françon A, Foote AM, Genty C, Rolland C, Roques CF, Bosson JL.Spa therapy in the treatment of knee OA: a large randomised multicentre trial. Ann Rheum Dis. 2010 ;69:660-5.

2 Tubach F, Ravaud P, Baron G, Falissard B, Logeart I, Bellamy N, Bombardier C, Felson D, Hochberg M, van der Heijde D, Dougados M. Evaluation of clinically relevant changes in patient reported outcomes in knee and hip OA: the minimal clinically important improvement.Ann Rheum Dis. 2005;64:29-33.

3 Rat AC, Coste J, Pouchot J, Baumann M, Spitz E, Retel-Rude N, Le Quintrec JS, Dumont-Fischer D, Guillemin F. OAKHQOL: a new instrument to measure quality of life in knee and hip OA.J Clin Epidemiol. 2005 Jan;58:47-55.

4 Gauchard GC, Vançon G, Meyer P, Mainard D, Perrin PP.On the role of knee joint in balance control and postural

strategies: effects of total knee replacement in elderly subjects with knee OA.Gait Posture. 2010;32:155-60.

5 Altman R, Asch E, Bloch D, Bole G, Borenstein D, Brandt K, Christy W, Cooke TD, Greenwald R, Hochberg M, et al.

Development of criteria for the classification and reporting of OA. Classification of OA of the knee.

Diagnostic and Therapeutic Criteria Committee of the American Rheumatism Association.Arthritis Rheum. 1986;29:1039-49

6 Cicuttini FM, Spector TD.OA in the aged. Epidemiological issues and optimal management.Drugs Aging. 1995;6:409-20..

7 Le Pen C, Reygrobellet C, Gerentes I. Financial cost of OA in France. The "COART" France study. Joint Bone Spine. 2005;72:567-70.

8 Loeuillle D, Chary-Valckenaere I. Imagerie de l’arthrose. EMC (Elsevier, MassonSAS, Paris), Appareil locomoteur, 14-003C-30, 2008.

9 Van den Berg WB. OA year 2010 in review: pathomechanisms of OA.OA Cartilage.

2011;19:338-41.

10 Conaghan PG, Vanharanta H, Dieppe PA .Is progressive OA an atheromatous vascular disease?Ann Rheum Dis. 2005;64:1539-41.

11 Sharma L, Pai YC. Impaired proprioception and OA..Curr Opin Rheumatol. 1997 ;9:253-8..

12 Felson DT, Lohmander LS. Whither OA biomarkers? OA Cartilage 2009;17:419- 22.

13 Zhang W, Moskowitz RW, Nuki G, Abramson S, Altman RD, Arden N, Bierma-Zeinstra S, Brandt KD, Croft P, Doherty M, Dougados M, Hochberg M, Hunter DJ, Kwoh K, Lohmander LS, Tugwell P.OARSI recommendations for the management of hip and knee OA, Part II: OARSI evidence-based, expert consensus guidelines.OA Cartilage. 2008;16:137-62.

14. Tubach F, Ravaud P, Baron G, Falissard B, Logeart I, Bellamy N, Bombardier C, Felson D, Hochberg M, van der Heijde D, Dougados MEvaluation of clinically relevant states in patient reported outcomes in knee and hip OA: the patient acceptable symptom state. Ann Rheum Dis. 2005 ;64:34-7.

15 Bellamy N, Bell MJ, Goldsmith CH, Pericak D, Walker V, Raynauld JP, Torrance GW, Tugwell P, Polisson R. The effectiveness of hylan G-F 20 in patients with knee OA: an application of two sets of response criteria developed by the OARSI and one set developed by OMERACT-OARSI. OA Cartilage. 2005 ;13:104-10.

16 Rannou F, Coudeyre E, Ribinik P, Macé Y, Poiraudeau S, Revel M.Establishing recommendations for physical medicine and rehabilitation: the SOFMER methodology.Ann Readapt Med Phys. 2007;50:100-10.

17 Zhang W, Doherty M, Arden N, Bannwarth B, Bijlsma J, Gunther KP, Hauselmann HJ, Herrero-Beaumont G, Jordan K, Kaklamanis P, Leeb B, Lequesne M, Lohmander S, Mazieres B, Martin-Mola E, Pavelka K, Pendleton A, Punzi L, Swoboda B, Varatojo R, Verbruggen G, Zimmermann-Gorska I, Dougados MEULAR evidence based recommendations for the management of hip OA: report of a task force of the EULAR Standing Committee for International Clinical Studies Including Therapeutics (ESCISIT).; EULAR Standing Committee for International Clinical Studies Including Therapeutics (ESCISIT).Ann Rheum Dis. 2005 ;64:669-81..

18 Roddy E, Doherty M.Guidelines for management of OA published by the American College of Rheumatology and the European League Against Rheumatism: why are they so different?Rheum Dis Clin North Am. 2003 ;29:717-31..

19 Diagnostic and therapeutic management of common lumbago and sciatica of less than 3 months of duration. Recommendations of the ANAES. Agence Nationale d'Accréditation et d'Evaluation en Santé]. J Radiol. 2000;81:1665-6.

20 Verhagen AP, Bierma-Zeinstra SM, Cardoso JR, de Bie RA, Boers M, de Vet HC. Balneotherapy for rheumatoid arthritis.Cochrane Database Syst Rev. 2003;:CD000518.

21 Konrad K, Tatrai T, Hunka A, Vereckei E, Korondi I.Controlled trial of balneotherapy in treatment of low back pain. Ann Rheum Dis. 1992 ;51:820-2.

22 Guillemin F, Constant F, Collin JF, Boulange M.Short and long-term effect of spa therapy in chronic low back pain.Br J Rheumatol. 1994 ;33:148-51.

23 Constant F, Collin JF, Guillemin F, Boulangé M. Effectiveness of spa therapy in chronic low back pain: a randomized clinical trial.J Rheumatol. 1995;22:1315-20.

24 Constant F, Guillemin F, Collin JF, Boulangé M. Use of spa therapy to improve the quality of life of chronic low back pain patients.Med Care. 1998 ;36:1309-14.

25 Elkayam O, Ophir J, Brener S, Paran D, Wigler I, Efron D, Even-Paz Z, Politi Y, Yaron M. Immediate and delayed effects

of treatment at the Dead Sea in patients with psoriatic arthritis.Rheumatol Int. 2000;19:77-82.

26 Franke A, Reiner L, Pratzel HG, Franke T, Resch KL. Long-term efficacy of radon spa therapy in rheumatoid arthritis—a randomized, sham-controlled study and follow-up.Rheumatology . 2000 ;39:894-902.

27 Buskila D, Abu-Shakra M, Neumann L, Odes L, Shneider E, Flusser D, Sukenik S. Balneotherapy for fibromyalgia at the Dead Sea.Rheumatol Int. 2001;20:105-8.

28 Neumann L, Sukenik S, Bolotin A, Abu-Shakra M, Amir M, Flusser D, Buskila D.The effect of balneotherapy at the Dead Sea on the quality of life of patients with fibromyalgia syndrome.Clin Rheumatol. 2001;20:15-9.

29 Evcik D, Kizilay B, Gökçen E. The effects of balneotherapy on fibromyalgia patients.Rheumatol Int. 2002;22:56-9.

30 Van Tubergen A, Landewé R, van der Heijde D, Hidding A, Wolter N, Asscher M, Falkenbach A, Genth E, Thè HG, van der Linden S. Combined spa-exercise therapy is effective in patients with ankylosing spondylitis: a randomized controlled trial. Arthritis Rheum. 2001;45:430-8.

31 Van Tubergen A, Boonen A, Landewé R, Rutten-Van Mölken M, Van Der Heijde D, Hidding A, Van Der Linden S.Cost effectiveness of combined spa-exercise therapy in ankylosing spondylitis: a randomized controlled trial.Arthritis Rheum. 2002 15;47:459-67.

32 Van Tubergen A, Hidding A.Spa and exercise treatment in ankylosing spondylitis: fact or fancy?Best Pract Res Clin Rheumatol. 2002 ;16:653-66.

33 Forestier R, Françon A, Saint Arroman F, Bertolino C, Graber-Duvernay B, Guillemot A, Slikh M.Are SPA therapy and pulsed electromagnetic field therapy effective for chronic neck pain? Randomised clinical trial. Second part: medicoeconomic approach].Ann Readapt Med Phys. 2007;50:148-53.

34 Forestier R, Françon A, Saint-Arromand F, Bertolino C, Guillemot A, Graber-Duvernay B, Slikh M, Duplan B.Are SPA therapy and pulsed electromagnetic field therapy effective for chronic neck pain? Randomised clinical trial First part: clinical evaluation].Ann Readapt Med Phys. 2007;50:140-7.

35 Forestier R, Françon A.Crenobalneotherapy for limb OA: systematic literature review and methodological analysis.Joint Bone Spine. 2008;75:138-48.

36 Forestier R.Magnitude and duration of the effects of two spa therapy courses on knee and hip OA: an open prospective study in 51 consecutive patients. Joint Bone Spine. 2000;67:296-304.

37 Wigler I, Elkayam O, Paran D, Yaron M. Spa therapy for gonarthrosis: a prospective study.Rheumatol Int. 1995;15:65-8.

38 Sukenik S, Flusser D, Abu-Shakra M. The role of spa therapy in various rheumatic diseases.Rheum Dis Clin North Am. 1999 ;25:883-97.

39 Guillemin F, Virion JM, Escudier P, De Talancé N, Weryha G.Effect on OA of spa therapy at Bourbonne-les-Bains. Joint Bone Spine. 2001;68:499-503.

40 Kovács I, Bender T. The therapeutic effects of Cserkeszölö thermal water in OA of the knee: a double blind, controlled, follow-up study.Rheumatol Int. 2002;21:218-21.

41 Tishler M, Rosenberg O, Levy O, Elias I, Amit-Vazina M.The effect of balneotherapy on OA. Is an intermittent regimen effective?Eur J Intern Med. 2004 ;15:93-96.

42 Yilmaz B, Goktepe AS, Alaca R, Mohur H, Kayar AH. Comparison of a generic and a disease specific quality of life scale to assess a comprehensive spa therapy program for knee OA.Joint Bone Spine. 2004;71:563-6.

43 Lee HJ, Park HJ, Chae Y, Kim SY, Kim SN, Kim ST, Kim JH, Yin CS, Lee H.Tai Chi Qigong for the quality of life of patients with knee OA: a pilot, randomized, waiting list controlled trial.Clin Rehabil. 2009;23:504-11.

44 Chalmers TC, Smith H Jr, Blackburn B, Silverman B, Schroeder B, Reitman D, Ambroz A.A method for assessing the quality of a randomized control trial.Control Clin Trials. 1981;2:31-49.

45 Koes BW, Bouter LM, van der Heijden GJ.Methodological quality of randomized clinical trials on treatment efficacy in low back pain.Spine (Phila Pa 1976). 1995 15;20:228-35.

46 Verhagen AP, de Vet HC, de Bie RA, Kessels AG, Boers M, Knipschild PG.Balneotherapy and quality assessment: interobserver reliability of the Maastricht criteria list and the need for blinded quality assessment.J Clin Epidemiol. 1998 ;51:335-41.

47 Van Tulder M, Furlan A, Bombardier C, Bouter L; Editorial Board of the Cochrane Collaboration Back Review Group.Updated method guidelines for systematic reviews in the cochrane collaboration back review group.Spine (Phila Pa 1976). 2003 15;28:1290-9.

48 unz R, Oxman AD. The unpredictability paradox: review of empirical comparisons of randomised and non-randomised clinical trials.BMJ. 1998;317:1185-90.

49 Moher D, Jones A, Lepage L; CONSORT Group (Consolitdated Standards for Reporting of Trials).Use of the CONSORT statement and quality of reports of randomized trials: a comparative before-and-after evaluation.JAMA. 2001 18;285:1992-5.

50 Moher D, Pham B, Jones A, Cook DJ, Jadad AR, Moher M, Tugwell P, Klassen TP.Does quality of reports of randomized trials affect estimates of intervention efficacy reported in meta-analyses?

Lancet. 1998 22;352:609-13.

51 Zelen protocols.Lancet. 1992 Jun 27;339(8809):1574-5.

52 Korvick JA, Peacock JE Jr, Muder RR, Wheeler RR, Yu VL.Addition of rifampin to combination antibiotic therapy for Pseudomonas aeruginosa bacteremia: prospective trial using the Zelen protocol.Antimicrob Agents Chemother. 1992;36:620-5.

53 Schellings R, Kessels AG, ter Riet G, Sturmans F.The Zelen design may be the best choice for a heroin-provision experiment.J Clin Epidemiol. 1999;52:503-7

54Armstrong PW, Watts DG.Clinical trials:randomization before consent. Biomedicine;1981;34:65-6.

55 Adamson J, Cockayne S, Puffer S, Torgerson DJ.Contemp. Review of randomised trials using the post-randomised consent (Zelen's) design. Clin Trials. 12.2006 ;27:305-19.

56 Rydevik K, Fernandes L, Nordsletten L, Risberg MA. Functioning and disability in patients with hip OA with mild to moderate pain.. J Orthop Sports Phys Ther. 2010 ;40:616-24.

57.Pagani CH, Böhle C, Potthast W, Brüggemann GP.Short-term effects of a dedicated knee orthosis on knee adduction moment, pain, and function in patients with OA. Arch Phys Med Rehabil.2010 ;91:1936-41

58. Kennedy DM, Stratford PW, Robarts S, Gollish JD.Using outcome measure results to facilitate clinical decisions the first year after total hip arthroplasty. J Orthop Sports Phys Ther. 2011 ;41:232-9

59 Abbott JH, Robertson MC, McKenzie JE, Baxter GD, Theis JC, Campbell AJ MOA Trial team.Exercise therapy, manual therapy, or both, for OA of the hip or knee: a factorial randomised controlled trial protocol.; Trials. 2009 Feb 8;10:11.

60 Bellamy N, Buchanan WW, Goldsmith CH, Campbell J, Stitt LW.Validation study of WOMAC: a health status instrument for measuring clinically important patient relevant outcomes to antirheumatic drug therapy in patients with OA of the hip or knee.J Rheumatol. 1988 ;15:1833-40.

61 Dworkin RH, Peirce-Sandner S, Turk DC, McDermott MP, Gibofsky A, Simon LS, Farrar JT, Katz NP. OA Cartilage. Outcome measures in placebo-controlled trials of OA: responsiveness to treatment effects in the REPORT database.2011;19:483-92.

62 Huskisson EC.Measurement of pain.Lancet. 1974 9;2:1127-31.

63 The MOS 36-item short-form health survey (SF-36). I. Conceptual framework and item selection.

Ware JE Jr, Sherbourne CD..Med Care. 1992;30:473-83.

64 Fioravanti A, Giannitti C, Bellisai B, Iacoponi F, Galeazzi M.Efficacy of balneotherapy on pain, function and quality of life in patients with OA of the knee. Int J Biometeorol. 2011 May 15.

65 Baumann C, Rat AC, Mainard D, Cuny C, Guillemin F.Importance of patient satisfaction with care in predicting OA-specific health-related quality of life one year after total joint arthroplasty..Qual Life Res. 2011 Apr 30.

66 Rat AC, Baumann C, Guillemin F.National, multicentre, prospective study of quality of life in patients with OA of the knee treated with hylane G-F 20.Clin Rheumatol. 2011 Apr 12.
